# The Methyltransferase CcKmt3 Regulates Cell Wall Degradation Enzymes Activity to Enhance the Infection Process in *Cytospora chrysosperma*

**DOI:** 10.1111/mpp.70246

**Published:** 2026-04-01

**Authors:** Wenjun Song, Yicheng Li, Wenwen Li, Yonglin Wang

**Affiliations:** ^1^ State Key Laboratory of Efficient Production of Forest Resources, Beijing Key Laboratory for Forest Pest Control, College of Forestry Beijing Forestry University Beijing China

**Keywords:** cell wall‐degrading enzymes, *Cytospora chrysosperma*, epigenetic regulation, methyltransferase, poplar canker

## Abstract

Woody canker diseases cause devastating timber losses worldwide. Canker fungi exploit cell wall‐degrading enzymes (CWDEs) to promote plant infection, yet the underlying mechanism of the transcriptional regulation of CWDEs remains largely unknown. Using RNA‐seq analysis and biochemical assays, we show that the poplar pathogen *Cytospora chrysosperma* upregulates CWDEs during canker progression, especially increased secretion of laccases. The histone lysine methyltransferase Kmt3 (CcKmt3) is induced by *C. chrysosperma* infection. Loss of *CcKmt3* leads to a genome‐wide reduction in H3K36me3 methylation. The Δ*Cckmt3* strain presented hyphal growth defects, reduced production of hydrolytic enzymes, and impaired virulence. By combining ChIP‐seq with RNA‐seq, we demonstrated that CcKmt3 controls two hydrolases, *CcLac11* and *CcPme5*, for plant cell degradation and virulence in *C. chrysosperma* . Furthermore, we showed that CcKmt3 affects hyphal growth by regulating the fungal cell wall integrity pathway. Taken together, this study demonstrates that CcKmt3 regulates the transcriptional expression of genes related to CWDEs by modulating H3K36me3 levels. This regulation influences enzyme activity, ultimately impacting the degradation of the plant cell wall and the pathogenicity processes of the poplar canker fungus.

## Graphical abstract

Cell wall‐degrading enzymes of *Cytospora chrysosperma* play a crucial role in the infection process. Kmt3 directly regulates Lac11 expression via H3K36me3, thereby influencing laccase activity and affecting *C. chrysosperma* 's degradation of lignocellulose.

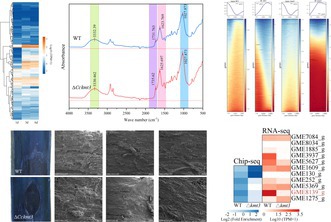

## 1 Introduction

Poplars constitute a pivotal component within forest ecosystems, especially planted forests. Poplars are constantly under attack by diseases and pests (Hartmann et al. 2022); among them, bark cankers stand out as one of the most destructive and recalcitrant forest diseases impacting woody plants globally (Wu et al. 2025). *Cytospora chrysosperma* is the main causal agent of poplar canker (Lin et al. 2024). This fungus colonises cambium tissues and causes necrotic lesions on the bark, which leads to extensive bark degradation, canker on the trunk or twigs and even results in the death of whole trees. These stem‐infecting pathogens cause significant economic and ecological losses, but the molecular mechanism by which they breach lignified bark tissues—a unique infection barrier in woody hosts—remains poorly understood.

Plant cell walls serve as the primary defence against microbial invasion, forming a robust physical barrier composed of cellulose, hemicellulose, pectin, lignin and structural proteins (Rebaque et al. 2025; Rui and Dinneny 2020; Shikata et al. 2025; Yu et al. 2024; Zhang et al. 2021, 2025). In woody plants, lignin, a chemically complex and highly recalcitrant polymer, plays a crucial role in reinforcing cell walls and providing mechanical strength (Gallego‐Giraldo et al. 2018; Gill et al. 2018; Marriott et al. 2016; Singh et al. 2026). To overcome these barriers, pathogens secrete cell wall‐degrading enzymes (CWDEs), which target and dismantle plant cell wall components, enabling nutrient acquisition and infection propagation (Gamez‐Arjona et al. 2022; Qiu et al. 2025; Xiao et al. 2024). Genomic sequencing of various pathogens has revealed that CWDEs are critical virulence factors, particularly in fungi lacking specialised infection structures (Kubicek et al. 2014). Functional analyses have demonstrated that disruption or inhibition of CWDE activity often results in reduced fungal virulence and increased susceptibility to host defences (Li et al. 2024; Sabbadin et al. 2021; Yu et al. 2024). In woody pathogens, enzymes such as β‐glucosidases (EC 3.3.1.21), pectate lyases and polygalacturonases are essential for degrading lignin and pectin, which are major components of plant cell walls (Feng et al. 2023; Huang et al. 2021; Yu et al. 2018).

While the enzymatic roles of CWDEs and their regulation by conventional transcription factors have been extensively studied (Aro et al. 2001), the involvement of epigenetic mechanisms in modulating CWDE expression remains poorly understood. Epigenetic modifications, such as histone lysine methylation, have emerged as key regulators of gene expression in fungi (Ampntelnour et al. 2026; Biswas et al. 2025; Cao et al. 2022). Trimethylation of histone H3 at lysine 36 (H3K36me3), catalysed by SET domain methyltransferases, is a particularly important modification that influences transcriptional regulation and fungal pathogenicity (Gu et al. 2017; Lukito et al. 2021; Zhao et al. 2024). Despite these advances, the role of epigenetic mechanisms in regulating the pathogenicity of woody bark canker fungi remains largely unexplored, leaving a critical gap in our understanding of fungal virulence in woody tissue diseases.

In this study, we demonstrated that the expression of CWDEs is significantly upregulated in *C. chrysosperma* during poplar bark infection and that the CcKmt3‐mediated H3K36me3 of *CcLac11* plays a pivotal role in canker pathogenesis. Additionally, we show that lignin and pectin degradation are key virulence factors for *C. chrysosperma* . These findings provide new insights into the epigenetic regulation of CWDE expression and highlight the importance of CcKmt3 in bark degradation, advancing our understanding of the molecular mechanisms underlying canker pathogenesis in woody hosts.

## 2 Results

### 2.1 Upregulated Expression of CWDEs Is Strongly Associated With *C. chrysosperma* Infection

The histopathological changes in poplar branches after infection with *C. chrysosperma* were investigated using scanning electron microscopy (SEM). Compared with those of the mock‐inoculated branches, the surfaces of the infected poplar branches presented pronounced structural disruption (Figure S1a–f), suggesting significant necrosis of branches under treatment with *C. chrysosperma* . Further observation via Fourier transform infrared (FTIR) spectroscopy revealed distinct peaks at 1641 cm^−1^ and 1735 cm^−1^ between the fungal infection and mock‐inoculated groups (Figure S1g), that is, the C=C stretching vibration in lignin and the O‐H stretching vibration in pectin, respectively. In the infected samples, the intensities of both peaks were significantly lower than those in the mock‐infected samples, indicating that *C. chrysosperma* can effectively decompose lignin and pectin within the poplar cell walls during infection.

**FIGURE 1:**
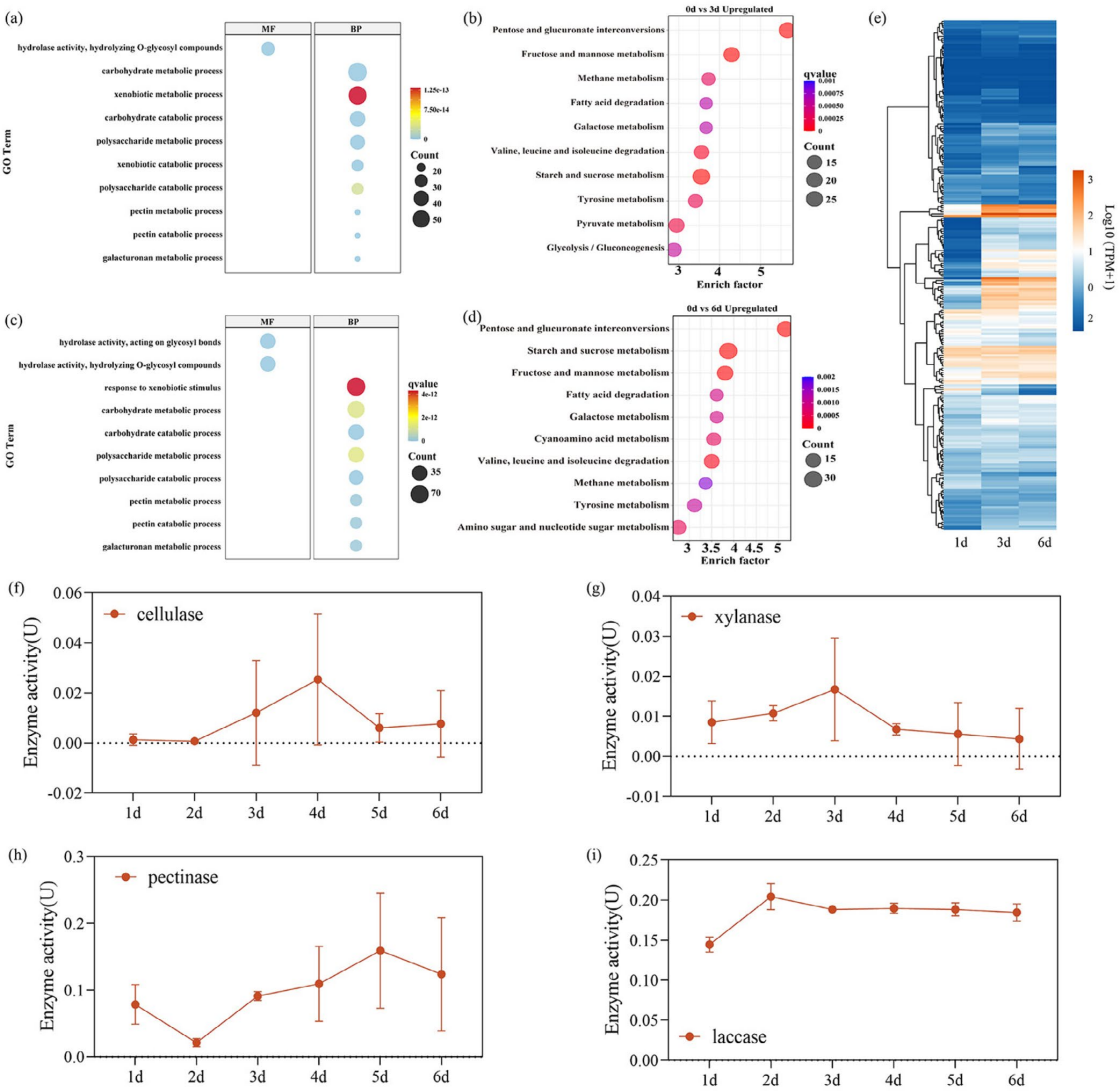
Transcriptional upregulation of cell wall‐degrading enzyme (CWDE) genes and enzymatic activity profiles during *Cytospora chrysosperma* infection. (a) GO enrichment analysis of differentially expressed genes (DEGs) in the 1 day‐post‐inoculation (dpi) versus 3 dpi comparison. (b) KEGG enrichment analysis of upregulated genes in the 1 dpi versus 3 dpi comparison. (c) GO enrichment analysis of DEGs in the 1 dpi versus 6 dpi comparison. (d) KEGG enrichment analysis of upregulated genes in the 1 dpi versus 6 dpi comparison. (e) Heatmap analysis of the main CWDEs. (f–i) Total cellulase, xylanase, pectinase and laccase activities during cultivation in water supplemented with 10% poplar bark extract.

To explore the molecular mechanisms underlying these alterations, transcriptional profiling of *C. chrysosperma* was conducted at 1, 3 and 6 days post‐inoculation (dpi). Comparative transcriptome analysis revealed 561 and 1032 differentially expressed genes (DEGs) at 3 and 6 dpi, respectively, relative to 1 dpi (Figure S2a–c). Among these genes, 1839 genes presented consistent expression patterns across infection stages, with 1335 genes upregulated and 504 genes downregulated (Figure S2d). Gene Ontology (GO) enrichment analysis revealed that the upregulated DEGs were significantly associated with carbohydrate metabolic processes and glycoside hydrolase activity (Figure 1a,b). Kyoto Encyclopedia of Genes and Genomes (KEGG) pathway analyses further revealed enrichment in metabolic pathways, including amino sugar and nucleotide sugar metabolism, pentose and glucuronate interconversions, fructose and mannose metabolism, and glycerolipid metabolism (Figure 1c,d).

Next, we aimed to identify the CWDEs secreted by *C. chrysosperma* that change the structure of the poplar cell wall during infection. We performed a cluster analysis of gene expression. This analysis revealed significant upregulation of genes encoding CWDEs during infection (Figure 1e). Enzymatic activity assays further demonstrated increased activities of cellulase, xylanase, pectinase and laccase during infection, with laccase and pectinase showing particularly high activity during the early stages (1–3 dpi) (Figure 1f–i). These findings suggest that *C. chrysosperma* induces significant structural damage to the poplar cell wall during infection, with the upregulation of key genes and increased enzyme activity involved in the degradation of cellulose, hemicellulose, pectin and lignin.

### 2.2 Disruption of CcKmt3 Function Leads to Defects in Cell Wall Integrity

**FIGURE 2:**
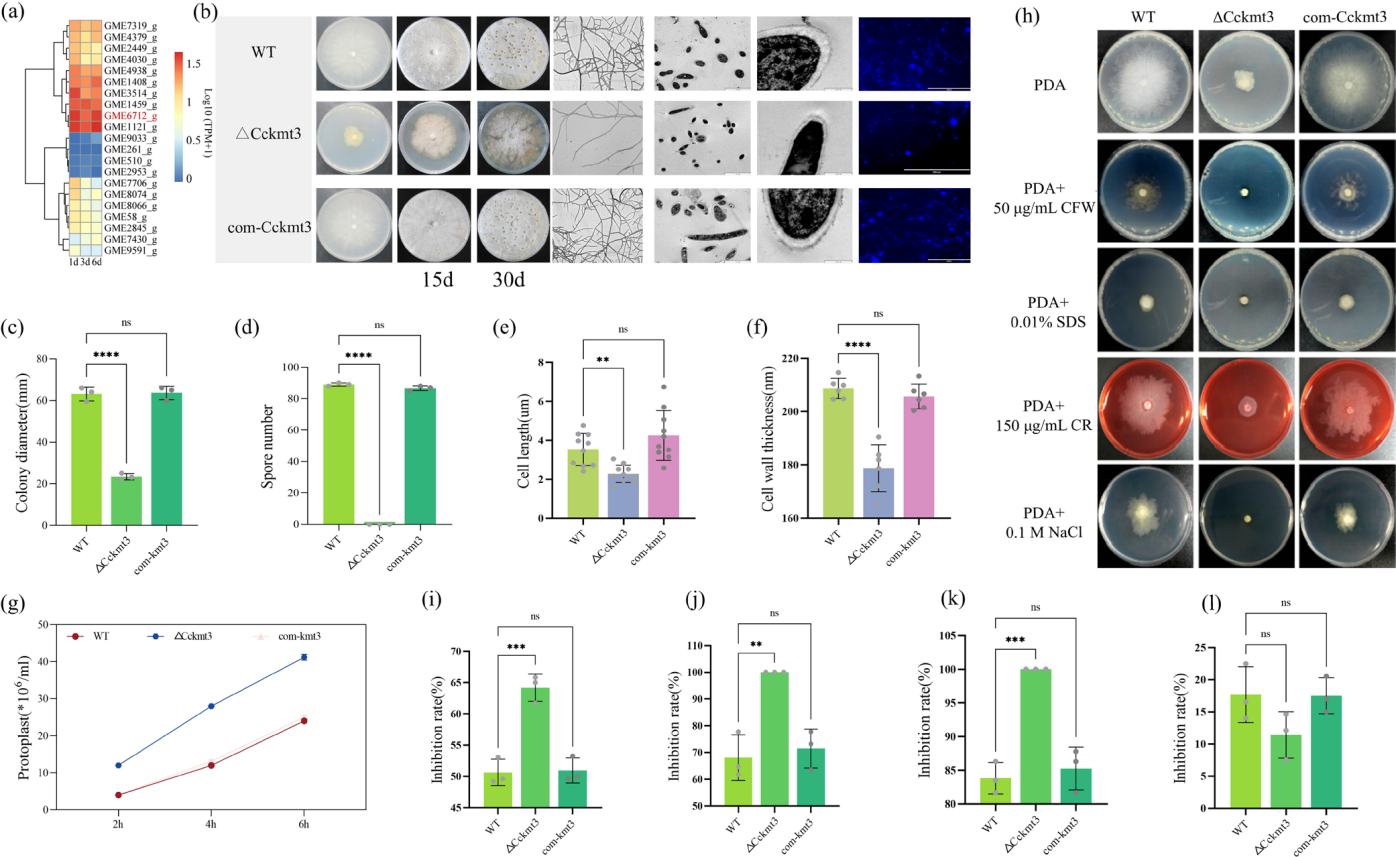
CcKmt3 is involved in hyphal growth and cell wall integrity. (a) Heatmap depicting the expression patterns of genes encoding methyltransferases in the *Cytospora chrysosperma* transcriptome during infection. (b) Compared with the wild‐type (WT) strain, the Δ*Cckmt3* strain presented significantly reduced hyphal growth. The WT, Δ*Cckmt3*, and com‐*kmt3* strains were cultured on potato dextrose agar (PDA) for 3 days, after which hyphal morphology was examined via light microscopy (scale bar 200 μm) and transmission electron microscopy (scale bar for cells 10 μm, scale bar for walls 1 μm). Images were captured to assess the phenotypic differences among the strains. Right column shows hyphae stained with Calcofluor White (CFW, scale bar 200 μm). (c) Comparison of colony growth diameter. (d) Quantification of spore production in each strain. (e) Measurement of fungal cell length. (f) Quantification of cell wall thickness. The values are presented as the means ± SDs and were calculated from measurements taken at six locations within the mycelial cells. (g) Protoplast release assay for evaluating the number of protoplasts under cell wall‐degrading enzyme digestion. (h) Sensitivity of the strains to cell wall stress agents (CFW, SDS, Congo Red [CR], and NaCl). The indicated strains were grown on PDA plates supplemented with 50 μg/mL CFW, 0.01% SDS, 150 μg/mL CR, or 0.1 M NaCl. (i–l) Statistical analysis of the growth inhibition ratios of strains cultured on media supplemented with CFW, SDS, CR, and NaCl. The error bars represent the SDs of three independent replicates. Student's *t* test, **p < 0.01, ***p < 0.001, ****p < 0.0001, ^ns^ *p* > 0.05.

While the regulation of CWDEs by transcription factors is well documented (Aro et al. 2001), the role of epigenetic regulators in this process is still unclear. Histone methylation, a key chromatin modification, is known to influence transcriptional activity. For the infection transcriptome data, the expression profiles of the histone methyltransferase gene family were examined. Among the 21 genes, *CcKmt3* (GME6712_g) was significantly induced (Figure 2a).

We functionally characterised CcKmt3 by obtaining mutant lines in which *CcKmt3* was disrupted (Δ*Cckmt3*) and SET domain deletion strains (*Cckmt3* ^ΔSET^). Comparative analysis of hyphal growth revealed that the Δ*Cckmt3* mutants presented a pronounced growth defect on solid media compared with the wild‐type (WT) strain (Figure 2b,c), and the quantity of spores produced was significantly reduced (Figure 2d). Microscopic observation further revealed a significant reduction in hyphal branching in Δ*Cckmt3* (Figure 2b). Compared with those of the WT strain, the hyphal cells of the Δ*Cckmt3* mutant were notably smaller, with thinner cell walls (Figure 2e,f). Calcofluor White (CFW) staining revealed a significant reduction in fluorescence intensity in the apical regions of the Δ*Cckmt3* mycelia (Figure 2b), indicating altered cell wall composition. Additionally, protoplast release assays demonstrated that the Δ*Cckmt3* mutant was more readily converted to protoplasts under the action of fungal CWDEs, suggesting compromised cell wall integrity (Figure 2g). Further analysis of cell wall stress responses revealed that the Δ*Cckmt3* mutant presented increased sensitivity to cell wall stressors, including CFW and SDS (Figure 2h–l). These results highlight the essential role of CcKmt3 in maintaining cell wall integrity during fungal growth and infection.

### 2.3 CcKmt3 Is Essential for Fungal Virulence and Plant Cell Wall Degradation

**FIGURE 3:**
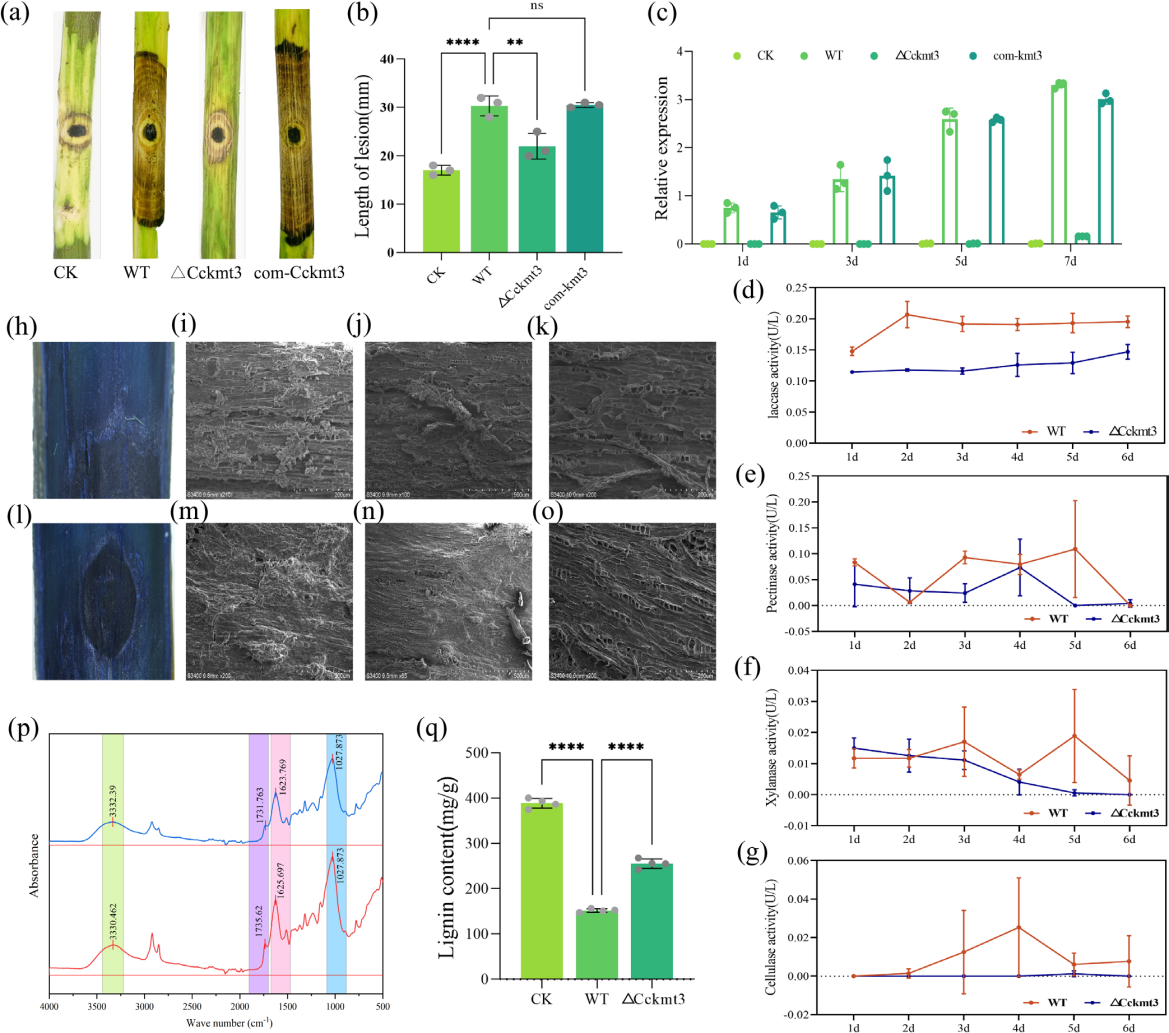
CcKmt3 is involved in the virulence and degradation of plant cell walls during interactions between *Cytospora chrysosperma* and its host. (a) Pathogenicity assay of the wild‐type (WT), Δ*Cckmt3* and complemented com‐*kmt3* strains on poplar branches. CK, mock inoculation. (b) Measurement of lesion lengths on branches at 6 days post‐inoculation (dpi). Student's *t* test, **p < 0.01, ****p < 0.0001, ^ns^ *p* > 0.05. (c) Quantification of the fungal biomass in the poplar leaves of the strains at 1–6 dpi. (d–g) Total laccase, pectinase, xylanase and cellulase activities during WT and Δ*Cckmt3* canker progression (at 1–6 dpi). (h) Trypan blue staining of the lesion site following infection with the WT strain. (i–k) Scanning electron microscopy (SEM) images of lesion sites following infection with the WT strain. (i) Interface between diseased and healthy tissue; (j) surface of healthy bark; (k) surface of the lesion site. (l) Trypan blue staining of the lesion site following infection with the Δ*Cckmt3* strain. (m–o) SEM images of lesion sites following infection with the Δ*Cckmt3* strain. (m) Interface between diseased and healthy tissue; (n) surface of healthy bark; (o) surface of the lesion site. (p) Fourier transform infrared spectra of plant cell wall components from branches treated with the WT (blue line) and Δ*Cckmt3* strains (red line) during infection. Pink shading indicates C=C stretching in lignin; purple shading indicates O‐H stretching in pectin. (q) Statistical analyses of lignocellulose content in mock, Δ*Cckmt3*, and WT strains. ****p < 0.0001.

To determine the role of CcKmt3 in the virulence of *C. chrysosperma* , pathogenicity assays were performed on poplar branches inoculated with mycelial plugs of the WT, Δ*Cckmt3* mutant and complemented com‐*kmt3* strains. Compared with both the WT and com‐*kmt3* strains, the Δ*Cckmt3* mutant caused significantly shorter lesions (Figure 3a,b), indicating a reduction in virulence. Consistent with this observation, the fungal biomass quantified within lesions induced by the Δ*Cckmt3* mutant was significantly lower than that in lesions caused by the WT strain (Figure 3c). Interestingly, although the growth of the Δ*Cckmt3* mutant was significantly greater than that of the WT strain on potato dextrose agar (PDA) plates, its growth and extension normalised when the incubation period was extended to 3–30 days. To further investigate the role of CcKmt3 in pathogenicity, lesion lengths caused by the Δ*Cckmt3* strain were measured at 10, 15 and 30 days, revealing that the pathogenicity of the CcKmt3‐deficient strain remained significantly reduced throughout the infection period (Figure S3a). Taken together, these results indicate that CcKmt3 is essential for maintaining fungal virulence during infection and suggest that its role in pathogenicity is not solely attributable to its effects on the growth of *C. chrysosperma* .

**FIGURE 4:**
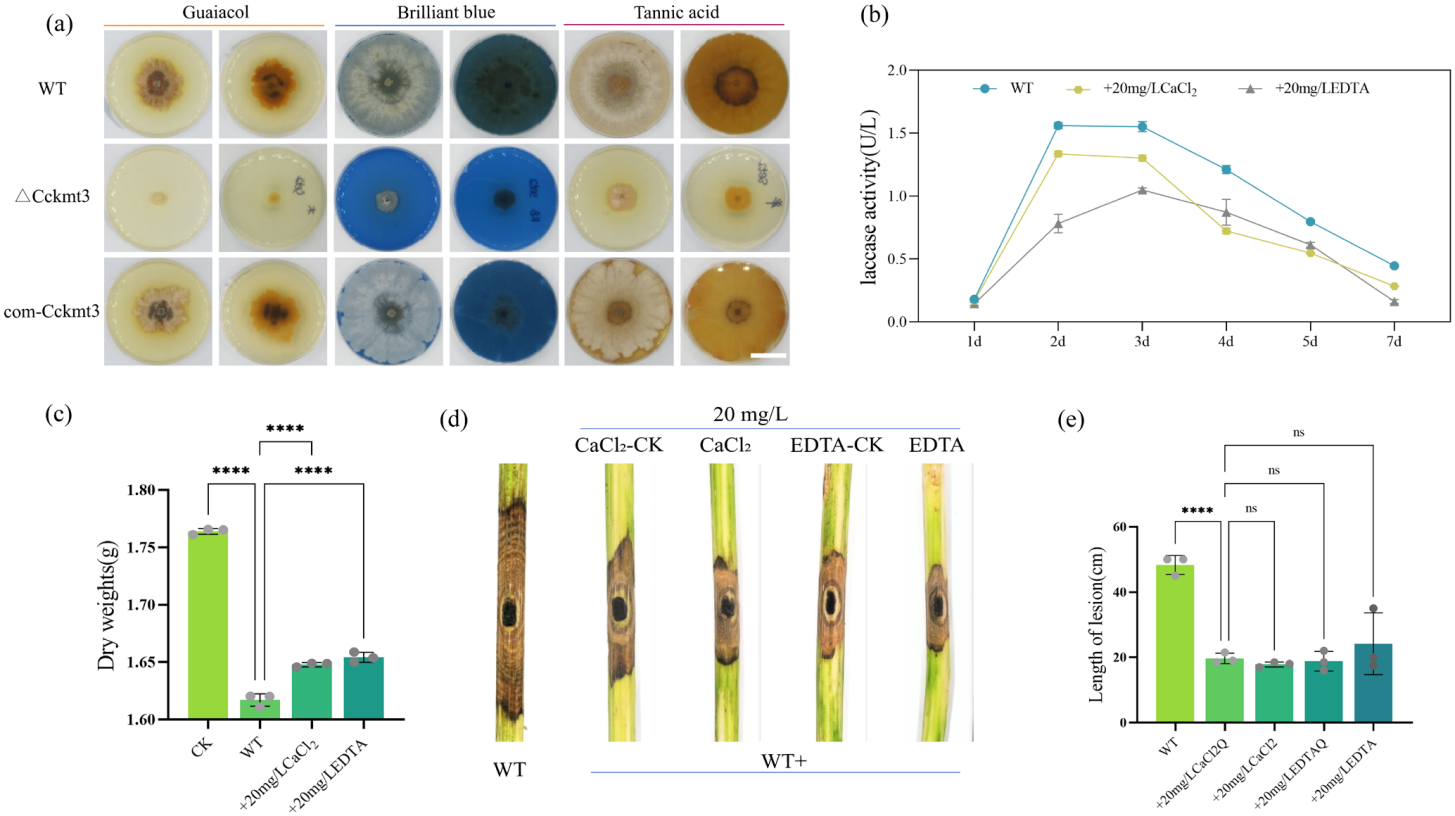
CcKmt3 regulates laccase activity, which plays a critical role in the pathogenicity of *Cytospora chrysosperma* . (a) Lignocellulose degradation by the wild‐type (WT), Δ*Cckmt3* mutant and complemented com‐*kmt3* strains grown on potato dextrose agar (PDA) plates supplemented with guaiacol, Remazol Brilliant Blue R or tannic acid. Images were taken after 20 days of incubation. (b) Comparison of laccase activity with or without treatment with a laccase inhibitor (CaCl_2_ or EDTA). (c) Analysis of poplar bark degradation in the presence and absence of laccase inhibitor treatment. CK, mock‐inoculation. (d) Pathogenicity assay showing lesion length on poplar bark with and without laccase inhibitor treatment. CaCl_2_‐CK and EDTA‐CK were sprayed with the treatment 1 day before inoculation with the WT strain, CaCl_2_ and EDTA were sprayed with the treatment 2 days after inoculation. Images were taken 6 days after inoculation. (e) Statistical analysis of lesion length in response to laccase inhibitor treatment. The data are presented as the mean ± SD from three independent biological replicates. Statistical significance was determined based on Student's *t* test (^ns^ *p* > 0.05, ****p < 0.0001).

The role of CcKmt3 in plant cell wall degradation was investigated by measuring the enzymatic activities of cellulase, xylanase, pectinase and laccase in the WT and Δ*Cckmt3* mutant strains during simulated infection over a 1–6‐day period (Figure 3d–g). The assays revealed a significant reduction in laccase and pectinase activities in the Δ*Cckmt3* mutant strain compared with the WT strain, indicating impaired enzymatic degradation of lignin and pectin. SEM analysis of poplar branches infected with the WT or Δ*Cckmt3* strains at 6 dpi revealed distinct morphological changes, with the Δ*Cckmt3* mutant exhibiting reduced tissue degradation compared with the WT (Figure 3h–o). FTIR spectroscopy further revealed significantly lower peak intensities corresponding to lignin and pectin functional groups in tissues infected with the Δ*Cckmt3* mutant than in those infected with the WT strain (Figure 3p). Consistently, lignin quantification at lesion sites revealed significantly higher lignin levels in lesions caused by the Δ*Cckmt3* mutant than in those caused by the WT strain (Figure 3q). Similarly, experiments involving the addition of lignin monomers, including guaiacol, Remazol Brilliant Blue R, and tannic acid, demonstrated that the Δ*Cckmt3* strain exhibited a markedly diminished capacity to degrade and utilise lignin monomers (Figure 4a). Together, these results demonstrate that CcKmt3 is essential for efficient fungal‐mediated degradation of lignin and pectin, which are critical components of the plant cell wall.

Next, we investigated the role of laccase in lignin degradation and pathogenicity. WT strains were treated with the laccase inhibitors EDTA and CaCl_2_. Inhibitor treatment significantly reduced laccase activity (Figure 4b) and resulted in a markedly diminished capacity to degrade poplar bark (Figure 4c). Pathogenicity inoculation assays of poplar branches revealed that, compared with the untreated control strain, the inhibitor‐treated strains produced significantly smaller lesions (Figure 4d,e). These results collectively demonstrate that laccase is essential for lignin degradation and plays a critical role in the pathogenicity of *C. chrysosperma* .

### 2.4 CcKmt3 Is the Master Regulator of CWDE Expression

**FIGURE 5:**
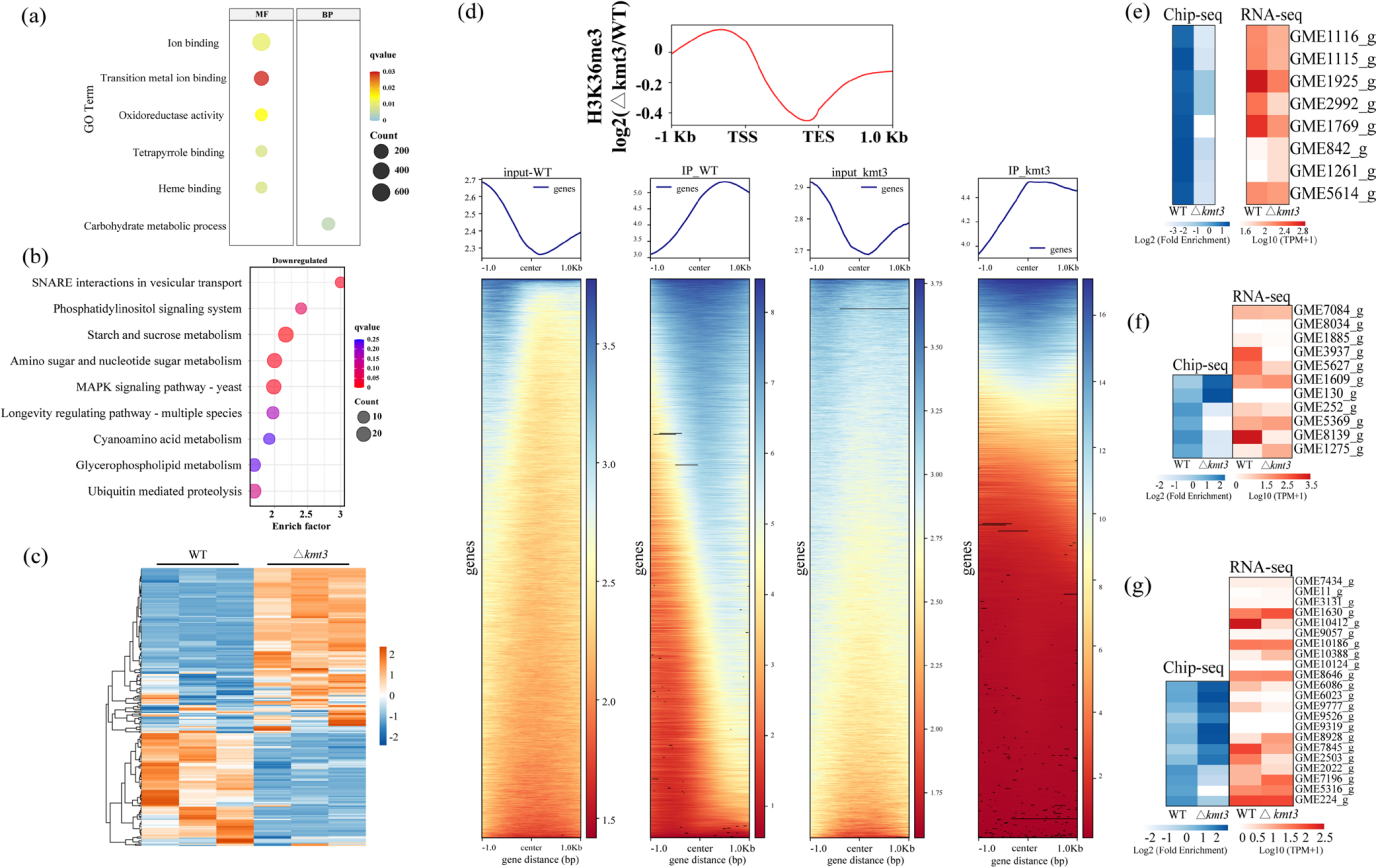
Altered transcriptional profiles and epigenetic modifications in the Δ*Cckmt3* strain compared with the wild‐type (WT) strain. (a) GO enrichment analysis of genes downregulated in the Δ*Cckmt3* strain. (b) KEGG pathway enrichment analysis of genes downregulated in the Δ*Cckmt3* strain. (c) Cluster analysis of cell wall‐degrading enzyme (CWDE) genes in the Δ*Cckmt3* strain revealed changes in expression profiles compared with those of the WT strain. (d) H3K36me3 enrichment levels in differential DiffBind peaks (−1 to +1 kb) in the WT and Δ*Cckmt3* strains. The *y*‐axis indicates H3K36me3 density, calculated as log_2_ (Δ*Cckmt3*/WT). TSS, transcription start site; TES, transcription end site. (e) Heatmaps displaying the H3K36me3 enrichment density (left) and corresponding transcriptional profiles (right) of the transcription factor *CcRlm1* (GME2992_g) and seven chitinase‐encoding genes (*CcCsh5*, GME1116_g; *CcCsh6*, GME1115_g; *CcCsh3*, GME1925_g; *CcCsh1*, GME1769_g; *CcCsh2*, GME842_g; *CcCsh7*, GME1261_g; *CcCsh4*, GME5614_g). (f) Heatmaps showing the H3K36me3 enrichment density (left) and corresponding transcriptional profiles (right) of laccase‐encoding genes. (g) Heatmaps presenting the H3K36me3 enrichment density (left) and corresponding transcriptional profiles (right) of pectinase‐encoding genes.

To investigate the role of CcKmt3 in transcriptional regulation and fungal pathogenicity, RNA sequencing (RNA‐seq) was performed on the WT and Δ*Cckmt3* mutant strains, revealing significant transcriptomic changes caused by the knockout of *CcKkmt3* (Figure S4a,b). GO enrichment analysis of the DEGs (*p* < 0.01) revealed that the downregulated DEGs were associated primarily with carbohydrate metabolic processes and cell wall organisation (Figure 5a), whereas KEGG pathway analysis revealed notable alterations in several metabolic pathways (Figure 5b). Notably, the Δ*Cckmt3* strain presented significant downregulation of genes associated with carbohydrate‐active enzymes (Figure 5c). GO enrichment analysis further revealed that genes involved in fungal cell wall synthesis were significantly affected by the deletion of *CcKmt3* (Figure 5e). Reverse transcription–quantitative PCR (RT‐qPCR) further confirmed the regulatory role of CcKmt3 in the transcription of these genes (Figure S4c). These findings indicate that CcKmt3 plays a key regulatory role in the expression of genes that are crucial for the pathogenicity of *C. chrysosperma* .

To further investigate the role of CcKmt3 in transcriptional regulation, we performed chromatin immunoprecipitation (ChIP)‐seq analysis to examine the genome‐wide distribution of H3K36me3 histone modifications in the WT and Δ*Cckmt3* strains. Compared with the WT strain, the Δ*Cckmt3* mutant presented a significant reduction in H3K36 trimethylation density (Figure S4f and Figure 5d). ChIP‐seq analysis of genes encoding chitinase‐related proteins enriched in the transcriptome revealed that the transcription of *CcRlm1* (a transcription factor regulating chitinase gene expression) and chitinase‐related genes (*CcCsh1/3/4/5/6*) is directly modulated by H3K36me3 modification (Figure 5e). Chitinase activity assays confirmed a notable decrease in enzyme activity in the Δ*Cckmt3* mutant (Figure S4b). Previous studies (Xie et al. 2023) have established the critical role of chitinases and chitin synthesis in fungal hyphal development and pathogenicity, highlighting the key role of H3K36me3 modification in the regulation of *C. chrysosperma* pathogenesis.

To better understand the connection between H3K36me3 modifications and gene regulation, RNA‐seq and ChIP‐seq analyses of the CWDE family revealed that among the 89 genes transcriptionally regulated by CcKmt3, 25 were marked with H3K36me3 modifications (Figure S4g and Table S2). Furthermore, combined RNA‐seq and ChIP‐seq analysis of the laccase‐encoding gene family identified *CcLac11* (GME8139_g) as the gene exhibiting the most pronounced transcriptional regulation by CcKmt3 (Figure 5f and Figures S4e and S5a), highlighting its potential importance in the regulatory network. However, no direct regulation by H3K36me3 modification was observed for *CcPme5* (GME7845_g), the pectinesterase family gene showing the most significant transcriptional change (Figure 5g and Figures S4e and S7a).

### 2.5 CcLac11 Is Essential for Lignin Degradation and Virulence

**FIGURE 6:**
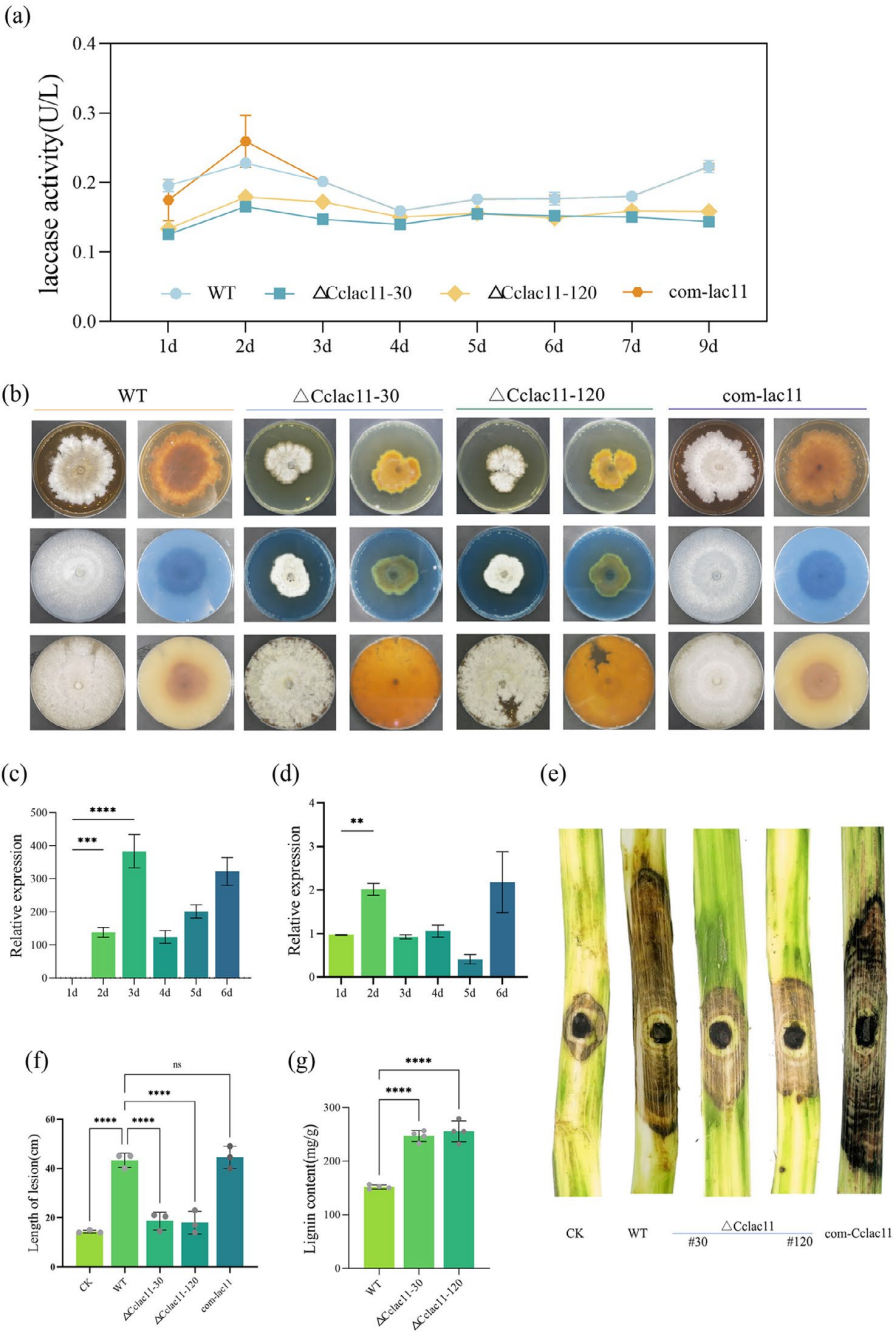
CcLac11 is associated with laccase activity, lignin degradation, and virulence in *Cytospora chrysosperma*. (a) Total laccase activity of the wild‐type (WT), Δ*Cclac11* and complemented com‐*lac11* strains during cultivation in water supplemented with 10% poplar bark extract. (b) Impaired lignocellulose degradation in the Δ*Cclac11* strain. The WT, Δ*Cclac11* and com‐*lac11* strains were grown on potato dextrose agar supplemented with guaiacol (top row), Remazol Brilliant Blue R (middle row) or tannic acid (bottom row), and images were captured after 20 days. (c) Reverse transcription‐quantitative PCR (RT‐qPCR) analysis of *CcLac11* expression during infection of poplar leaves with *C. chrysosperma* . (d) RT‐qPCR analysis of *CcLac11* expression during incubation in water supplemented with 10% poplar bark extract. (e) Pathogenicity assay comparing the WT, Δ*Cclac11* and com‐*lac11* strains on poplar branches. CK, mock‐inoculation. (f) Measurement of lesion lengths on infected poplar branches at 6 days post‐inoculation. (g) Statistical analysis of lignocellulose content in the WT and Δ*Cclac11* strains during *C. chrysosperma* infection. A quantitative analysis of lignocellulose content was performed to compare the WT and Δ*Cclac11* strains during infection. Student's *t* test, **p < 0.01, ***p < 0.001, ***p < 0.0001, ^ns^ *p* > 0.05.

Sequence analysis of CcLac11, a member of the laccase enzyme family, revealed the presence of a Cu oxidase domain and a signal peptide (Figure S5b), indicating that CcLac11 is a secreted laccase. To investigate its functional role, we generated *CcLac11* deletion mutants. Compared with the WT strain, the Δ*Cclac11* mutant presented a significant reduction in extracellular laccase activity (Figure 6a). Consistent with this, chromogenic assays using PDA supplemented with guaiacol, Remazol Brilliant Blue R or tannic acid demonstrated that, compared with the WT strain, the Δ*Cclac11* strain exhibited markedly reduced lignin monomer degradation (Figure 6b). These findings suggest that CcLac11 is an important contributor to the secreted laccase activity of *C. chrysosperma* .

Expression analysis revealed that *CcLac11* was significantly upregulated during the early stages of infection, highlighting its potential role in fungal pathogenicity (Figure 6c,d). Pathogenicity assays further confirmed this hypothesis, as the Δ*Cclac11* mutant displayed significantly reduced virulence on poplar branches compared with the WT (Figure 6e,f). Quantification of lignin content at lesion sites revealed that lesions caused by Δ*Cclac11* infection contained significantly higher lignin levels than those induced by the WT strain (Figure 6g). The complementation of the Δ*Cclac11* strain with the full‐length *CcLac11* gene restored laccase activity and virulence to WT levels, confirming the essential role of CcLac11 in fungal pathogenicity. Furthermore, overexpression of *CcLac11* effectively reversed the defects in fungal pathogenicity observed in *C. chrysosperma* (Figure S5c,d). These findings provide strong evidence that CcKmt3 regulates fungal pathogenicity at the transcriptional level through its downstream target, the laccase CcLac11.

**FIGURE 7:**
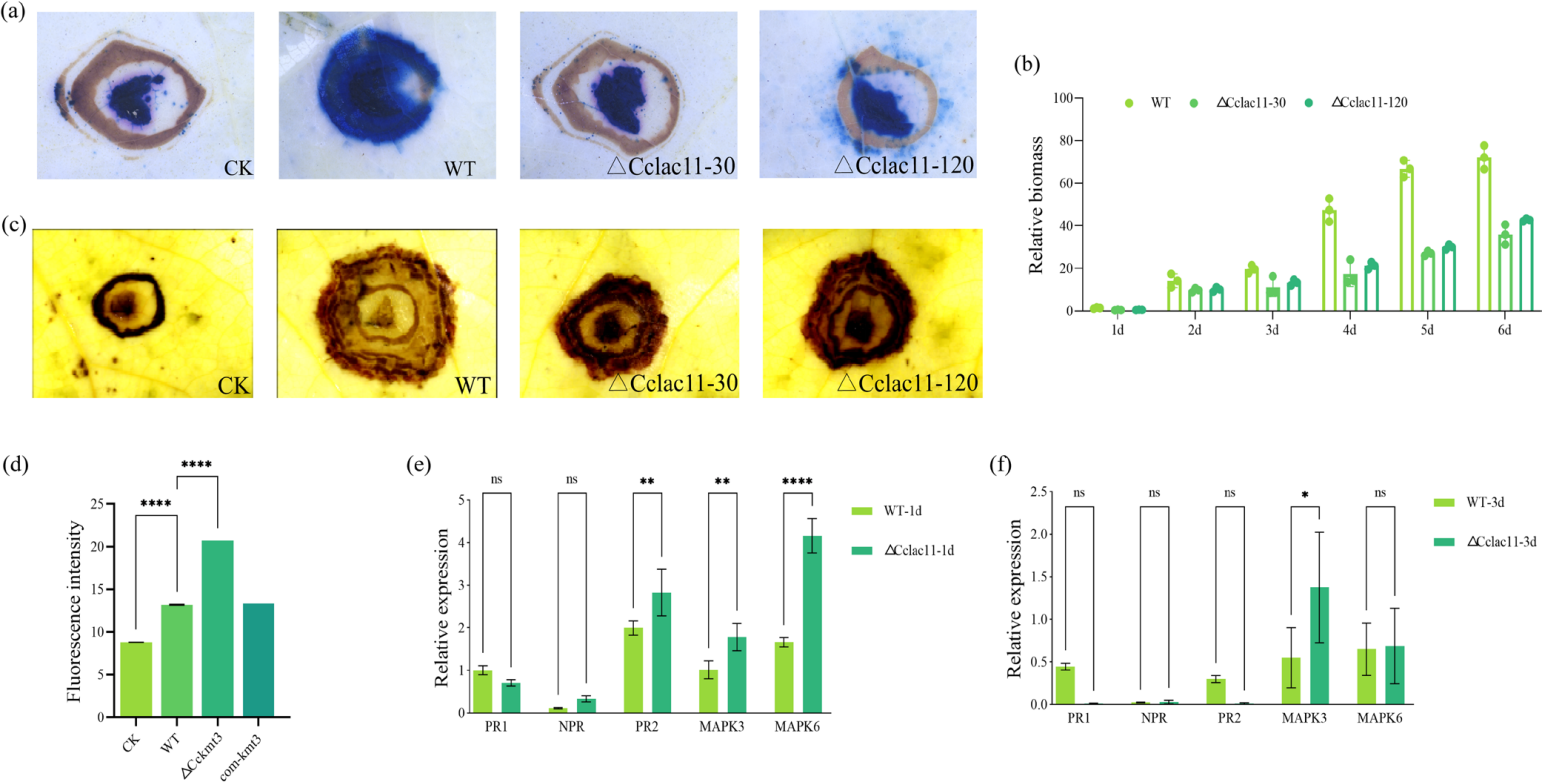
Differences in host defence responses following infection by the wild‐type (WT) and Δ*Cclac11* strains. (a) Microscopic observation of poplar epidermal cells infected with the WT and Δ*Cclac11* strains stained with 0.4% trypan blue. Intact cells appear transparent, whereas cells with disrupted membranes are stained blue, indicating cell damage. CK, mock‐inoculation. (b) Quantification of fungal biomass in poplar leaves infected with the WT and Δ*Cclac11* strains from 1 to 6 days post‐inoculation (dpi). (c) Reactive oxygen species (ROS) accumulation in the leaves of poplar plants inoculated with the WT and Δ*Cclac11* strains, visualised by staining with 3,3′‐diaminobenzidine (DAB). (d) Quantification of DAB staining intensity in infected poplar leaves via ImageJ software. (e, f) Expression levels of pathogenesis‐related (*PR* *1*, *PR2*), nonexpressor of pathogenesis‐related (*NPR*), and mitogen‐activated protein kinase (*MAPK* *3*, *MAPK6*) genes in poplar branches at 1 dpi (e) and 3 dpi (f) following inoculation with the WT and Δ*Cclac11* strains, as analysed via reverse transcription‐quantitative PCR. Student's *t* test, *p < 0.05, **p < 0.01, ****p < 0.0001, ^ns^ *p* > 0.05.

To further understand the impact of CcLac11 deletion on fungal infection, we performed trypan blue staining and fungal biomass analysis of poplar bark (Figure 7a,b). These assays revealed a substantial reduction in fungal biomass in the Δ*Cclac11* strain compared with the WT strain. Additionally, plant defence responses were analysed following infection with the Δ*Cclac11* mutants. Reactive oxygen species (ROS) burst assays revealed that, compared with the WT strain, the Δ*Cclac11* mutant elicited a significantly stronger ROS response (Figure 7c,d). Furthermore, RT‐qPCR analysis of poplar pathogenesis‐related genes revealed that the expression of *PcPR2*, *PcMAPK3* and *PcMAPK6* was strongly induced upon infection with the Δ*Cclac11* strain (Figure 7e,f). Notably, both the Δ*Cclac11* mutant and the strain treated with laccase inhibitors exhibited normal filamentous growth under in vitro conditions (Figures S5e,f and S6), suggesting that CcLac11‐mediated laccase activity is specifically required for *C. chrysosperma* virulence without affecting the in vitro growth of the fungus.

### 2.6 CcPme5 Is Important for Pectin Degradation and Virulence

**FIGURE 8:**
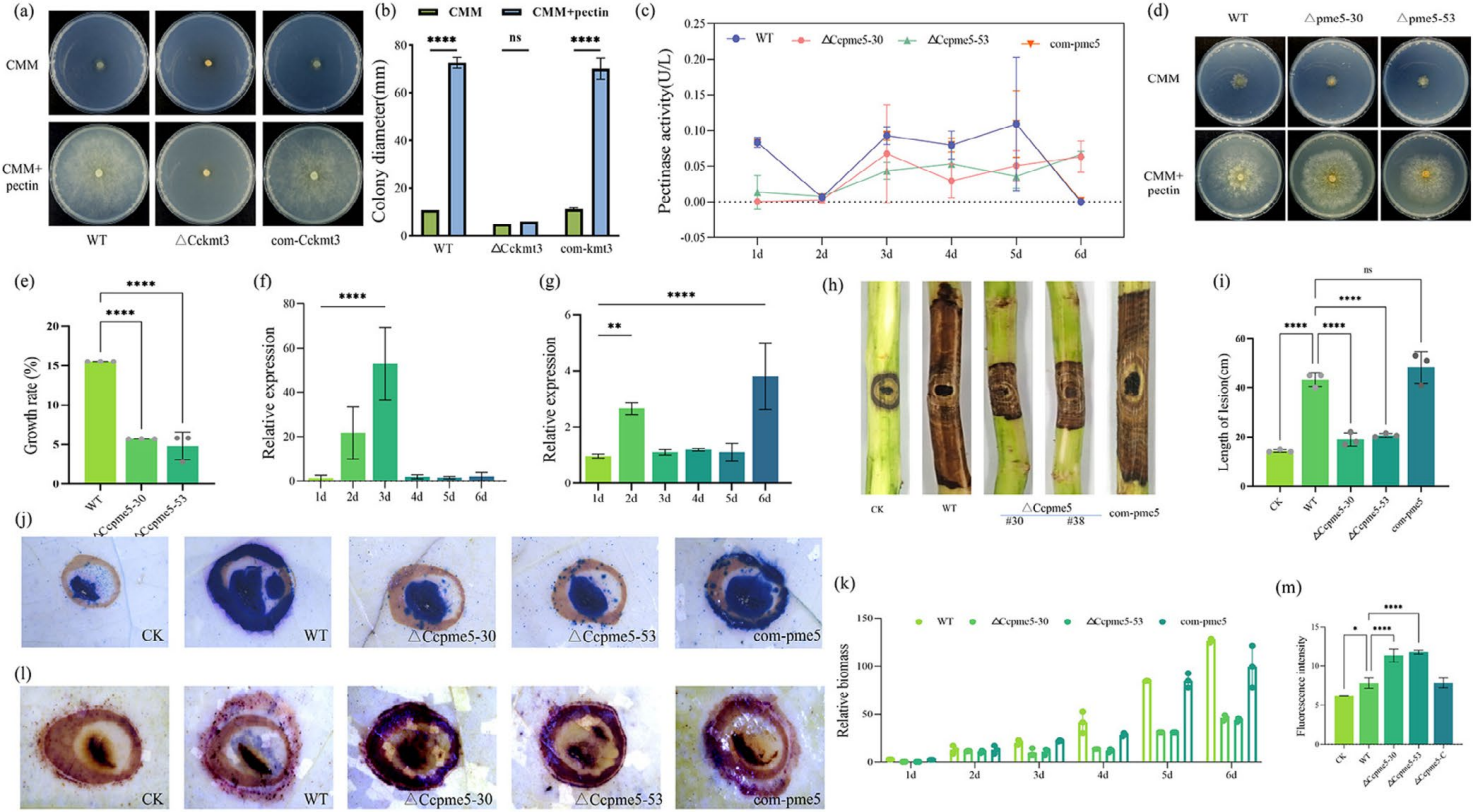
CcKmt3 regulates pectinase activity and modulates the transcription of the pectin methylesterase family gene *CcPme5*, which contributes to pectinase activity and virulence in *Cytospora chrysosperma* . (a) Colony morphology of the wild‐type (WT), Δ*Cckmt3* and complemented com‐*kmt3* strains grown on carbon‐free minimal medium (CMM) with or without 1% pectin supplementation. Images were captured after 10 days of incubation. (b) Statistical analysis of hyphal growth in the WT, Δ*Cckmt3* and com‐*kmt3* strains after 10 days of incubation, with measurements expressed as the mean ± standard deviation. (c) Total pectinase activity of the WT, Δ*Ccpme5*, and com‐*pme5* strains during cultivation in water supplemented with 10% poplar bark extract. (d) Colony morphology of the WT and Δ*Ccpme5* strains grown on CMM with or without 1% pectin supplementation. Images were captured after 10 days of incubation. (e) Statistical analysis of the pectin degradation capacity of the WT and Δ*Ccpme5* strains. (f) Reverse transcription‐quantitative PCR (RT‐qPCR) analysis of *CcPme5* expression in poplar leaves during infection by *C. chrysosperma* . (g) RT‐qPCR analysis of *CcPme5* expression during incubation in water supplemented with 10% poplar bark extract. (h) Pathogenicity assay comparing the virulence of the WT, Δ*Ccpme5* and com‐*pme5* strains on poplar branches. CK, mock‐inoculation. (i) Measurement of lesion lengths on infected branches at 6 days post‐inoculation (dpi), with statistical analysis of differences among strains. (j) Microscopic observation of poplar epidermal cells infected with the WT, Δ*Ccpme5* and com‐*pme5* strains stained with 0.4% trypan blue. (k) Quantification of fungal biomass in poplar leaves infected with the WT, Δ*Ccpme5*, and com‐*pme5* strains from 1 to 6 dpi via qPCR. (l) Accumulation of reactive oxygen species (ROS) in poplar leaves inoculated with the WT, Δ*Ccpme5* and com‐*pme5* strains, visualised by staining with 3,3′‐diaminobenzidine (DAB). (m) The intensity of DAB staining in infected poplar leaves was quantified via ImageJ software, and the differences between the strains were statistically analysed. Student's *t* test, *p < 0.05, **p < 0.01, ****p < 0.0001, ^ns^ *p* > 0.05.

CcKmt3 plays a crucial role in pectin degradation by significantly influencing the enzymatic activity of pectinases and the efficiency of pectin utilisation (Figure 8a). RNA‐seq analysis revealed that CcKmt3 affected the expression of *CcPme5* (Figure 5g), a key member of the pectin methylesterase family, suggesting an indirect regulatory role of CcKmt3 in pectinase activity. To investigate the function of CcPme5, a Δ*Ccpme5* mutant was generated. The Δ*Ccpme5* mutant exhibited normal filamentous growth under in vitro conditions (Figure S7b,c). Compared with the WT strain, this mutant exhibited significantly reduced extracellular pectinase activity (Figure 8c and Figure S7d) and impaired growth on both solid and liquid minimal media where pectin served as the sole carbon source (Figure 8d,e). These results confirmed the essential role of CcPme5 in pectin degradation.

Expression analysis revealed that *CcPme5* was upregulated during the early stages of infection (Figure 8f,g), indicating that it contributes to fungal pathogenicity. Pathogenicity assays revealed that, compared with the WT strain, the Δ*Ccpme5* mutant caused significantly smaller lesions on poplar branches (Figure 8h,i), highlighting the importance of the virulence of CcPme5. Furthermore, overexpression of *CcPme5* effectively reversed the defects in fungal pathogenicity observed in *C. chrysosperma* (Figure S7e,f). The complementation of the Δ*Ccpme5* mutant with the full‐length *CcPme5* gene restored both pectinase activity and virulence to levels comparable to those of the WT strain, confirming the critical role of CcPme5 in fungal pathogenicity.

Further analyses, including trypan blue staining and fungal biomass quantification in infected poplar bark (Figure 8j,k), revealed a significant reduction in fungal biomass in the Δ*Ccpme5* mutant. Additionally, ROS burst measurements indicated that, compared with the WT strain, the Δ*Ccpme5* mutant elicited a stronger oxidative stress response in the host (Figure 8l,m), suggesting that CcPme5 contributes to mitigating host defences during infection. Collectively, these findings demonstrate that CcPme5 is a key virulence determinant in *C. chrysosperma* and that its expression is indirectly regulated by CcKmt3.

## 3 Discussion

The cell wall is a critical defensive structure for plants (Fan et al. 2024; Yang et al. 2024; Zhang et al. 2025; Zhao et al. 2026), serving as a physical barrier against potential microbial pathogens. CWDEs produced by pathogenic fungi are known to compromise the structural integrity of plant cell walls, thereby promoting the ability of the pathogen to invade and colonise the host (Xiao et al. 2024). Recent studies have highlighted the role of transcription factors in regulating the expression of genes encoding CWDEs (Aro et al. 2001). However, the mechanisms through which epigenetic modifications, particularly histone methylation, influence the transcriptional regulation of CWDEs remain poorly understood. This study identified CcKmt3 as a key regulator of transcription in *C. chrysosperma* . Specifically, CcKmt3 modulates the expression of the transcription factor *CcRlm1*, chitin synthase‐related genes, and the laccase family gene *CcLac11* through H3K36me3 histone methylation. These regulatory interactions impact chitin synthesis and laccase activity, ultimately influencing the ability of *C. chrysosperma* to expand lesions on poplar branches. These findings provide novel insights into the epigenetic regulation of fungal pathogenicity and underscore the importance of histone modifications in modulating fungus–host interactions.

KMT family genes have been shown to impair asexual growth and pathogenicity in *Fusarium* (Studt‐Reinhold et al. 2024; Tang et al. 2021). Consistent with these findings, CcKmt3 regulates fungal growth, sporulation and pathogenicity in *C. chrysosperma* (Figures 2 and 3). Further analysis of host responses revealed that, compared with the WT strain, the Δ*Cckmt3* mutant exhibited a significantly reduced ability to induce cell death in poplar cells (Figure S3b), accompanied by a greater ROS burst (Figure S3c,d). This increase in ROS production suggests that the absence of CcKmt3 triggers heightened oxidative stress responses in the host. Oxidative stress challenge experiments further demonstrated that CcKmt3 deficiency increases the susceptibility of *C. chrysosperma* to oxidative stress (Figure S3e), highlighting the critical role of CcKmt3 in fungal stress tolerance and its ability to mitigate host defence mechanisms during infection. Additionally, the Δ*Cckmt3* mutant presented significantly altered expression of effector genes (Figure S8). These findings emphasise the multifaceted role of CcKmt3 in fungal pathogenicity and its interaction with host defences, which is consistent with its reported functions in *Phytophthora sojae* (Chen et al. 2023). Taken together, the reduced pathogenicity observed in the Δ*Cckmt3* mutants is not solely attributable to defects in hyphal elongation but probably involves additional virulence factors regulated by CcKmt3.

The mechanisms underlying the establishment of H3K36me3 have been extensively studied in several model species (Bhattacharya et al. 2021; Kizer et al. 2005; Wagner and Carpenter 2012) but remain poorly understood in *C. chrysosperma* . Previous studies have demonstrated that the SRI domain plays a crucial role in H3K36me3 deposition by directly interacting with RNA polymerase II subunit B1, the largest subunit of RNA polymerase II (Kim and Lee 2023). The presence of an SRI domain in CcKmt3 suggests functional conservation of this mechanism in *C. chrysosperma* (Figure S3f). Our western blot analysis revealed that approximately 50% of H3K36me3 was retained in the *CcKmt3* knockout mutant (Figure S5f), indicating that H3K36me3 deposition was not completely abolished in the absence of CcKmt3. This partial retention suggests functional redundancy with other histone methyltransferases. Similar findings have been reported in filamentous fungi, where two homologues of SET2 contribute to H3K36me3 deposition (Bicocca et al. 2018; Janevska et al. 2018). In these fungi, double‐knockout mutants of the two homologues resulted in the complete loss of H3K36me2/me3, whereas single‐knockout mutants retained more than 20% of H3K36me3 (Gu et al. 2017; Janevska et al. 2018). Consistent with this, our RNA‐seq data detected transcriptional expression of other histone methyltransferase family members during infection, suggesting that additional KMT3 homologues may contribute to H3K36me3 deposition in *C. chrysosperma* . To fully eliminate H3K36me3 methylation, construction of a double‐knockout mutant targeting *Cckmt3* and other potential homologues may be necessary. Further investigation is required to identify and characterise these additional methyltransferases and to elucidate their specific roles in the epigenetic regulation of H3K36 methylation in *C. chrysosperma* . Such studies will provide deeper insights into the functional redundancy and complexity of histone methylation in this species.

In plant‐pathogenic fungi, the cell wall is crucial for survival, morphogenesis and virulence. As a dynamic protective barrier, it undergoes continuous synthesis and remodelling to evade plant recognition and adapt to environmental challenges during infection (Gow et al. 2017). CcKmt3 deficiency leads to slower growth, thinner cell walls, and increased sensitivity to stressors. Previous studies have shown that CcRlm1 binds directly to the *CcChs6* promoter to regulate gene expression. Additionally, *CcGna1* has been identified as a novel target of CcRlm1, and its deletion significantly reduces chitin content in the cell wall. Our ChIP‐seq analysis indicates that CcKmt3 targets H3K36me3 to regulate the transcription of *CcRlm1* and chitin synthase genes, affecting chitin synthase activity and compromising cell wall integrity. These findings underscore the pivotal role of CcKmt3 in maintaining cell wall integrity and highlight its potential as a target for controlling fungal pathogenicity.

Laccase, a secreted multicopper oxidase, has been identified as a key virulence factor in numerous plant pathogens, facilitating fungal penetration and colonisation (Dong et al. 2023; Westrick et al. 2024). In this study, we demonstrated that *C. chrysosperma* secretes substantial amounts of laccase during infection (Figure 1), which plays a pivotal role in lignin degradation within plant cell walls and contributes to fungal pathogenesis (Figure S1g). We found that H3K36me3 is essential for regulating laccase gene expression, with CcKmt3 directly modifying the chromatin structure of *CcLac11* to enhance transcription (Figure 5f). These results highlight the significance of epigenetic regulation in fungal pathogenicity and offer new insights into laccase‐mediated lignin degradation.

Pectin is a key structural component of plant cell walls, as demonstrated by chemical and genetic analyses (Shen et al. 2025). Pectate lyases, such as from *Fusarium sacchari* and *Verticillium dahliae*, play crucial roles in pectin degradation and virulence in fungi (Perotto et al. 1997; Wang et al. 2023; Yang et al. 2018). Pectin methylesterase (PME), a ubiquitous cell wall‐modifying enzyme present in both plants and microorganisms, also plays a crucial role in pectin metabolism. In this study, we demonstrated that substantial amounts of pectinase are secreted during infection, facilitating the degradation of pectin components in poplar cell walls. Functional analysis of the pectin methylesterase family gene *CcPme5* revealed its significant contribution to both pectinase activity and the pathogenicity of *C. chrysosperma* . Further investigation revealed that CcKmt3 strongly influences the expression of *CcPme5*. Interestingly, this regulation was independent of H3K36me3 modification, suggesting that alternative mechanisms, such as other histone marks or transcription factor‐mediated pathways, may control *CcPme5* expression. These findings provide valuable insights into the molecular basis of pectinase‐mediated cell wall degradation and underscore the complexity of the regulatory networks driving fungal pathogenicity.

While significant progress has been made in understanding the transcriptional regulation of CWDEs in plant pathogens (Aro et al. 2001), the role of epigenetic modifications in these regulatory mechanisms remains poorly understood. This study demonstrated that CcKmt3 directly modulates the transcription of the *CcLac11* gene through H3K36me3 modification, thereby regulating the laccase activity of *C. chrysosperma* . Increased laccase activity facilitates the degradation of poplar cell wall components, promoting fungal infection and colonisation. Despite these advances, key questions remain regarding the regulation of CWDE genes, including potential cross‐talk and feedback mechanisms between histone modifications and transcription factors. Addressing these questions will be critical for further elucidating the molecular basis of fungal pathogenicity. The findings presented here provide valuable insights into the epigenetic regulation of CWDEs and their role in fungal virulence, offering a foundation for the development of innovative strategies to manage fungal diseases in forest ecosystems and improve the control of plant pathogens.

## 4 Experimental Procedures

### 4.1 Fungal Strains and Targeted Deletion

The wild‐type strain of *C. chrysosperma* (CFCC 89981), which was isolated from *Populus* and sourced from the China Forestry Culture Collection Center, was used in this study. Fungal strains were cultivated on PDA at 25°C or in potato dextrose broth (PDB) with shaking at 150 rpm and 25°C.

Targeted deletion strains of *CcKmt3*, *CcLac11* and *CcPme5* were generated via the split‐marker method (Xie et al. 2023). The entire open reading frame of each gene was replaced by the hygromycin resistance gene. The upstream and downstream flanking sequences were amplified with primers (Table S1); then, both flanking fragments were fused to the hygromycin resistance gene and *CcKmt3* via fusion PCR. For the complementation of the targeted deletion strains, a fragment containing the entire coding region of each gene along with its native promoter and terminator regions was amplified via PCR with specific primers (Table S1). The polyethylene glycol‐mediated transformation method was carried out as described previously (Xie et al. 2023).

### 4.2 Vegetative Growth and Virulence Assays

For vegetative growth assays, PDA was inoculated with the WT strain, the Δ*Cckmt3* knockout mutant, or the complemented com‐*kmt3* transformant at 25°C in the dark. Colony diameters were measured 2 dpi. Additionally, growth assays for the *CcLac11* and *CcPme5* genes were conducted via the same methodology.

The virulence of *C. chrysosperma* was assessed in 1‐year‐old poplar branches (Li et al. 2025). Briefly, poplar branches were scalded with a hot iron rod measuring 5 mm in diameter and inoculated with 5 mm‐diameter fungal agar plugs, after which the infection was maintained for 6 days. When inoculating poplar leaves, gently create small burn marks on the leaf surface using a soldering iron, followed by the application of 5 mm‐diameter fungal agar plugs. A humid environment was maintained to facilitate infection development.

### 4.3 Western Blotting Assay

Five‐day‐old *C. chyrsosperma* mycelia collected from PDB were ground in liquid nitrogen. Next, 800 μL of lysis buffer (1% SDS in Tris‐EDTA buffer) was added to 100 mg of pulverised mycelium powder. The lysates were mixed by vortexing for 30 min at 4°C. Supernatants of protein samples were mixed with loading buffer (P0015, Beyotime) and denatured at 95°C for 10 min. Anti‐H3K36me3 (A20379, ABclonal) and anti‐H3 (AC070, ABclonal) were used as primary antibodies, and goat anti‐rabbit IRDye 800CW (Odyssey, no. 926‐32211; LI‐COR) was used as a secondary antibody. The signals were recorded by an Odyssey laser imaging system (LI‐COR) and quantified via ImageJ. The H3K36me3 level was calculated as the H3K36me3:H3 signal, and H3K36me3 levels were compared with those in the WT.

### 4.4 RNA Isolation and RT‐qPCR Analysis

Total RNA was isolated using TRIzol reagent (Invitrogen) and purified using a PureLink RNA Mini Kit (Invitrogen) according to the manufacturer's instructions. For RT‐qPCR assays, cDNA was synthesised and qPCR was performed using SuperReal PreMix Plus (Vazyme) on a 7500 Real‐Time PCR System (Applied Biosystems). The *CcActin* gene was used as an endogenous reference in both RT‐qPCR assays. Relative expression levels were calculated via the 2^−ΔΔ*C*t^ method. All primers used in this study are listed in Table S1.

### 4.5 Native ChIP‐Seq and RNA‐Seq

Mycelia were collected from 5‐day‐old fungal strains for ChIP‐seq and RNA‐seq analysis. The mycelia were then placed on filter paper and crushed in liquid nitrogen. Native ChIP experiments were performed as previously described (Wang et al. 2020), with cross‐linking performed with 1% formaldehyde and grinding in liquid nitrogen at the conclusion of the cross‐linking process. Chromatin preparations were sonicated into fragments ranging from 0.2 to 0.5 kkb. Antibodies specific for H3ac (Merck, catalogue no. 06‐599) and H3K36me3ac (Merck, catalogue no. 07‐352) were added to the chromatin, which was then purified with salmon sperm DNA/A‐agarose beads. The precipitates were subsequently eluted from the beads. The DNA was then recovered via a QIAquick spin column (Qiagen). The RNA‐seq libraries were prepared by Majorbio Company.

### 4.6 Measurement of Laccase and Pectinase Activities

Fungal strains were cultivated on PDA supplemented with 0.4 g/L guaiacol, Remazol Brilliant Blue R, or tannic acid. The plates were then incubated at 25°C for 20 days for colourimetric assessment. To assess enzyme activity during infection, these strains were added to liquid media supplemented with 100 g/L poplar bark and shaken at 25°C for 7 days. Samples were taken every 2 days and centrifuged at 12,000 *g* for 15 min to obtain the crude enzyme mixture, which was analysed according to a previously described method. Agar plugs 5 mm in diameter were taken. The mycelium was then transferred to minimal substrates containing 1% pectin as the sole carbon source for 5 days. Pectinase activity was quantified via the 3,5‐dinitrosalicylic acid (DNS) method (Wu et al. 2017).

To assess the lignin content in poplar branches following infection, 1‐year‐old poplar branches were inoculated with these strains. Agarose plugs were applied as a control group. The inoculated branches were incubated at 25°C for 7 days. The infected areas were subsequently scraped, dried at 80°C, and analysed via a lignin content determination kit (Sangon Biotech).

### 4.7 Observation via SEM or FTIR Spectroscopy

To examine the microstructure of the bark tissue following fungal infection, a field emission scanning electron microscope (SU8010M, Hitachi) was used. The samples were scanned at an accelerating voltage of 10 kV, and the morphology was recorded.

To assess changes in the material composition of tree bark after hot iron rod treatment and infection with fungal strains, bark samples from infected trees were collected, dried to a constant weight, and analysed via an FTIR spectrometer (Nicolet iS50, Thermo Scientific).

### 4.8 Determination of the Cell Wall Structure and Composition

Digital images were acquired using an FEI Tecnai G2 Twin transmission electron microscope equipped with an EMSIS Eagle digital camera to compare the cell wall architecture between the WT and Δ*Cckmt3* strains (Li et al. 2025). The cell size and cell wall thickness were quantified using ImageJ software.

To detect exposed chitin on the cell wall surface, hyphae were incubated with FITC‐labelled wheat germ agglutinin (WGA; 30 mg/mL, MKbio) for 20 min in the dark. For total chitin staining, hyphae were treated with CFW (20 mg/mL) for 15 min in the dark. The fluorescent signals were visualised via an Imager Z1 fluorescence microscope (Zeiss).

## Author Contributions

**Wenjun Song:** methodology, data curation, writing – original draft. **Yicheng Li:** formal analysis. **Wenwen Li:** visualisation. **Yonglin Wang:** writing – review and editing and resources.

## Funding

This work was supported by National Key Research and Development Program of China (2022YFD1401000) and the Fundamental Research Funds for the Central Universities (QNTD202510).

## Conflicts of Interest

The authors declare no conflicts of interest.

## Supporting information


**Figure S1:** Degradation of plant cell wall components during infection by *Cytospora chrysosperma* .

**Figure S2:** Transcriptomic analysis of differential gene expression during *Cytospora chrysosperma* infection.

**Figure S3:** Functional characterisation of CcKmt3 in chitinase activity, pathogenicity, host‐pathogen interaction, oxidative stress tolerance and protein domain structure during *Cytospora chrysosperma* infection.

**Figure S4:** Gene expression analysis and global H3K36me3 modification in *Cytospora chrysosperma* strains with altered CcKmt3 function.

**Figure S5:** Functional characterisation of CcLac11 and its role in fungal development, growth and pathogenicity in *Cytospora chrysosperma* .

**Figure S6:** Impact of laccase inhibitor treatment on mycelial growth in comparison to the untreated control.

**Figure S7:** Functional characterisation of CcPme5 and its role in fungal development, growth and pathogenicity in *Cytospora chrysosperma* .

**Figure S8:** Heatmaps showing the H3K36me3 enrichment density (left) and corresponding transcriptional profiles (right) of effector genes.

**Table S1:** Primers used in the study.

**Table S2:** Significant differences in gene expression levels of cell wall degradation enzyme families and peak analysis.

## Data Availability

The data used in this study have been submitted to the Genome Sequence Archive, which is publicly accessible at https://bigd.big.ac.cn/gsa/browse/CRA022602; https://ngdc.cncb.ac.cn/omix/release/OMIX015267; https://ngdc.cncb.ac.cn/omix/release/OMIX015268. The data supporting the findings of this study are presented in the figures, Figures [Link], [Link], and Tables S1 and S2.
