# Supplementary material for: The Methyltransferase CcKmt3 Regulates Cell Wall Degradation Enzymes Activity to Enhance the Infection Process in Cytospora chrysosperma
Source: Mol Plant Pathol. 2026 Apr 1;27(4):e70246. doi: 10.1111/mpp.70246 (PMC13045292; doi:10.1111/mpp.70246)
Supplement: Supplementary file 1 — Figure S1: Degradation of plant cell wall components during infection by Cytospora chrysosperma . [file MPP-27-e70246-s003.docx]

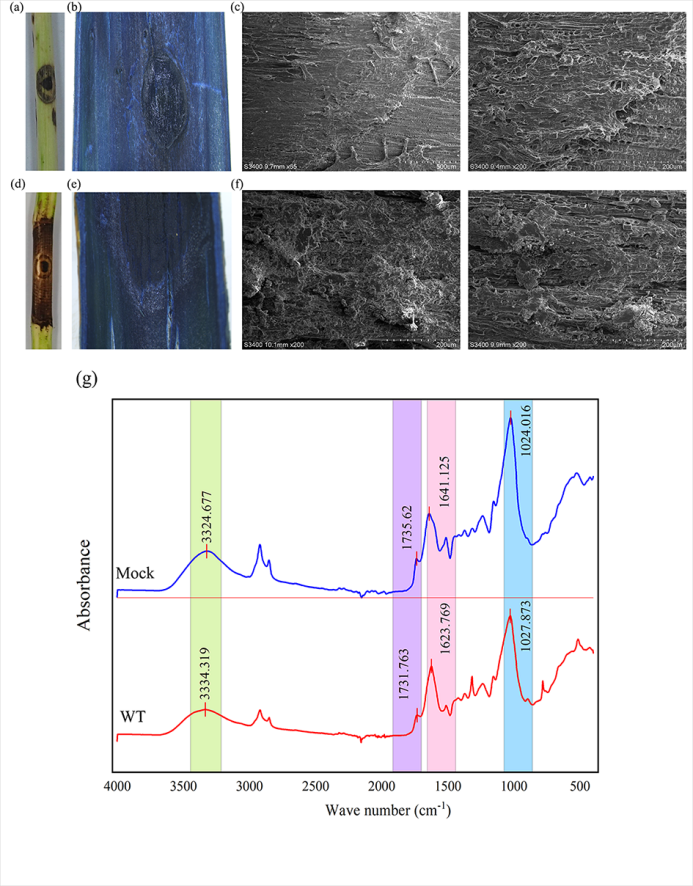


**Supplementary FIRGRE 1 Degradation of plant cell wall components during infection by *C. chrysosperma*.**

(a-c) SEM images of branches scalded with a 5 mm diameter hot iron rod.

(d-f) SEM images of *C. chrysosperma* under infection conditions.

(g) FT-IR spectra of plant cell wall components from branches treated with 5 mm diameter hot iron (Mock) and *C. chrysosperma* during infection (WT).
