# Supplementary material for: The Methyltransferase CcKmt3 Regulates Cell Wall Degradation Enzymes Activity to Enhance the Infection Process in Cytospora chrysosperma
Source: Mol Plant Pathol. 2026 Apr 1;27(4):e70246. doi: 10.1111/mpp.70246 (PMC13045292; doi:10.1111/mpp.70246)
Supplement: Supplementary file 2 — Figure S2: Transcriptomic analysis of differential gene expression during Cytospora chrysosperma infection. [file MPP-27-e70246-s005.docx]

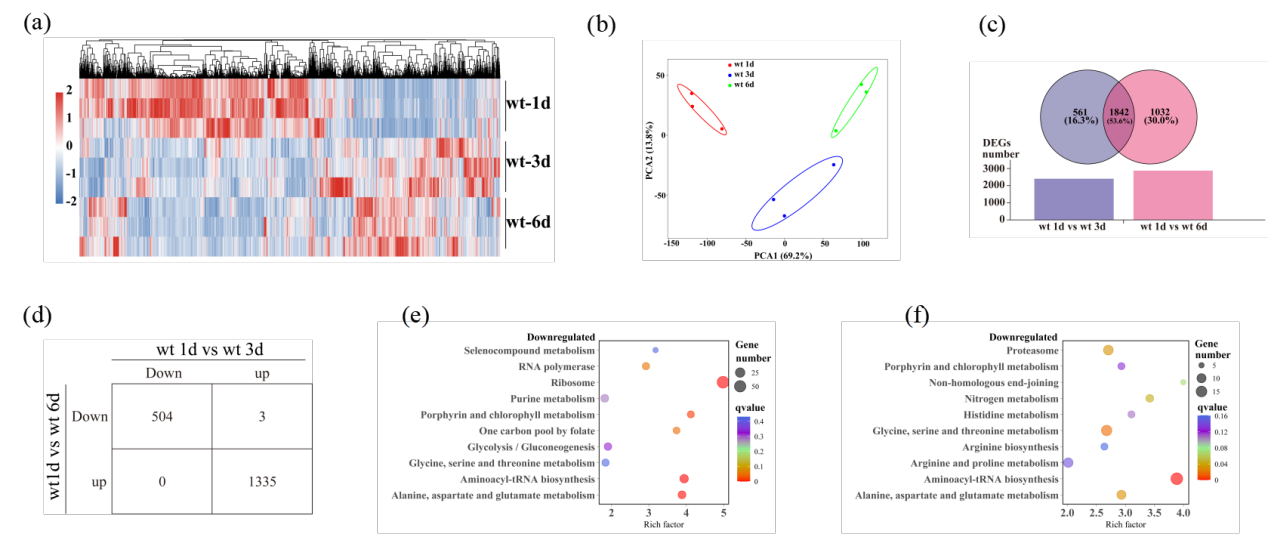


**Supplementary FIRGRE 2 Transcriptomic analysis of differential gene expression during *C. chrysosperma* infection.**

1. Cluster analysis of DEGs demonstrated consistent expression patterns across three biological replicates.
2. PCA highlighting intergroup differences and the reproducibility of samples within groups.
3. Statistical summary of upregulated and downregulated DEGs at 1 day, 3 days, and 6 days post-infection.
4. Venn diagram showing the number of DEGs associated with the 1 day, 3 day and 6 day processes.
5. KEGG enrichment analysis of downregulated genes comparing the 1-day and 3-day infection stages.
6. KEGG enrichment analysis of downregulated genes in the 1 day vs 6 day comparison.
