# Supplementary material for: The Methyltransferase CcKmt3 Regulates Cell Wall Degradation Enzymes Activity to Enhance the Infection Process in Cytospora chrysosperma
Source: Mol Plant Pathol. 2026 Apr 1;27(4):e70246. doi: 10.1111/mpp.70246 (PMC13045292; doi:10.1111/mpp.70246)
Supplement: Supplementary file 3 — Figure S3: Functional characterisation of CcKmt3 in chitinase activity, pathogenicity, host‐pathogen interaction, oxidative stress tolerance and protein domain structure during Cytospora chrysosperma infection. [file MPP-27-e70246-s010.docx]

*
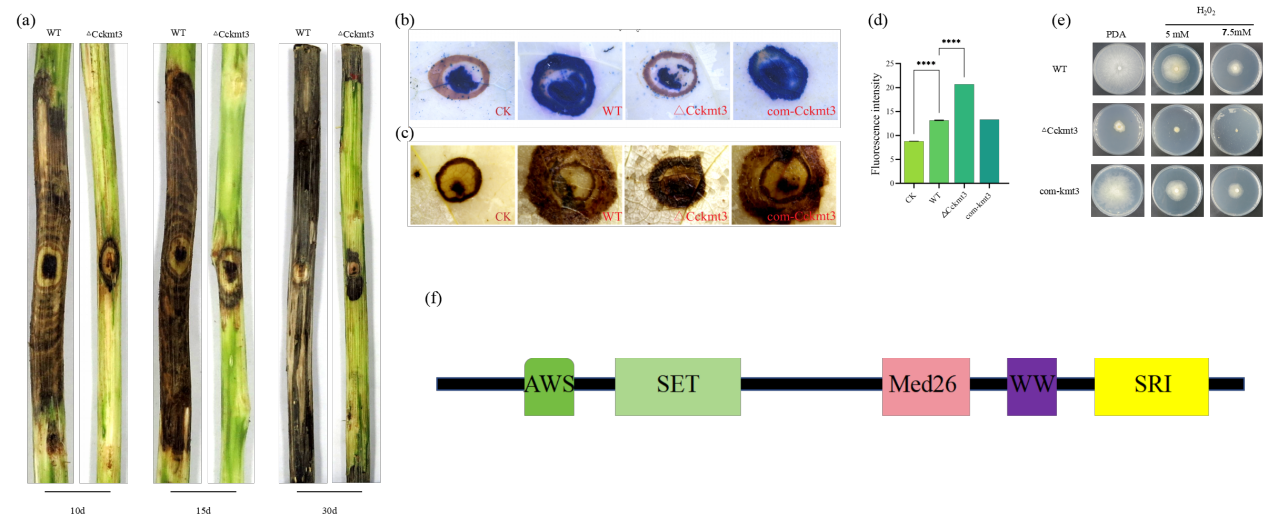
*

**Supplementary FIRGRE 3 Functional characterization of CcKmt3 in chitinase activity, pathogenicity, host-pathogen interaction, oxidative stress tolerance, and protein domain structure during *C. chrysosperma* infection.**

1. Pathogenicity assay of WT, ΔCckmt3 strains on poplar branches at 10dpi, 15dpi, and 30 dpi.
2. Microscopic observation of poplar epidermal cells infected with WT, Δ*Cckmt3* and com-kmt3 strains, stained with 0.4% trypan blue, revealed differences in host-pathogen interactions.
3. ROS accumulation in poplar leaves inoculated with WT, Δ*Cckmt3* and com-kmt3 strains, visualized as oxidative stress response.
4. Quantification of ROS intensity in infected poplar leaves using ImageJ software, with statistical analysis of differences between strains.
5. Sensitivity of fungal strains to oxidative stress. The indicated strains were cultured on plates supplemented with 5 mM or 7.5 mM H_2_O_2_, respectively, to assess their tolerance to oxidative stress.
6. Domain structure analysis of the CcKmt3 protein was conducted to identify functional regions associated with its activity.
