# Supplementary material for: The Methyltransferase CcKmt3 Regulates Cell Wall Degradation Enzymes Activity to Enhance the Infection Process in Cytospora chrysosperma
Source: Mol Plant Pathol. 2026 Apr 1;27(4):e70246. doi: 10.1111/mpp.70246 (PMC13045292; doi:10.1111/mpp.70246)
Supplement: Supplementary file 4 — Figure S4: Gene expression analysis and global H3K36me3 modification in Cytospora chrysosperma strains with altered CcKmt3 function. [file MPP-27-e70246-s007.docx]

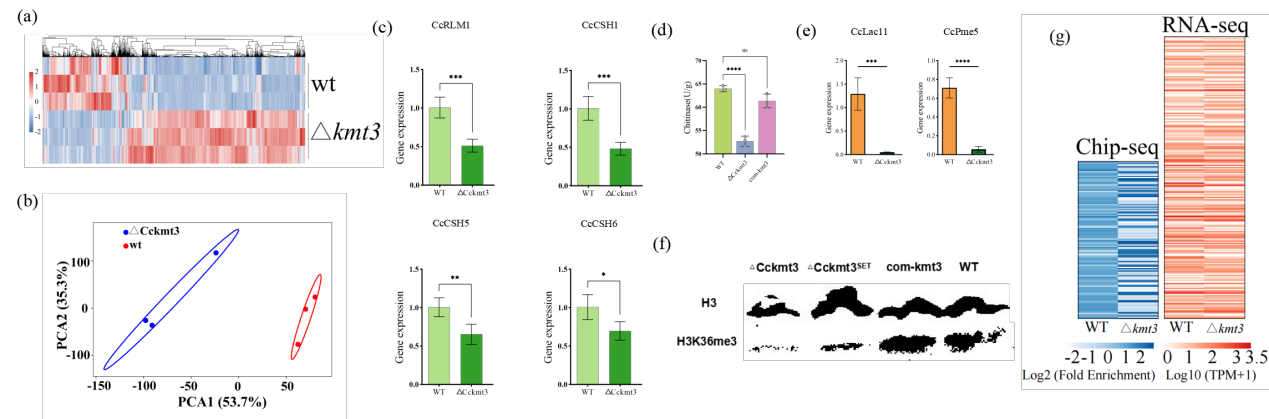


**Supplementary FIRGRE 4 Gene expression analysis and global H3K36me3 modification in *C. chrysosperma* strains with altered CcKmt3 function.**

1. Cluster analysis revealed that the patterns of DEGs were consistent across the three biological replicates for each strain.
2. PCA highlights the differences between the WT and Δ*Cckmt3* strains and confirms the reproducibility of the samples within each group.
3. qPCR analysis of *CcRlm1*, *CcCSH1*, *CcCSH5*, *CcCSH6* gene expression in the WT and Δ*Cckmt3* strains.
4. Chitinase activity assays were performed for WT, ΔCckmt3 and com-kmt3 to assess enzymatic activity.
5. qPCR analysis of *CcLac11* and *CcPme5* gene expression in WT and Δ*Cckmt3* strains.
6. Western blot analysis of global H3K36me3 levels across WT, Δ*Cckmt3*, com-*kmt3* and structural domain deletion strains (*Cckmt3*^ΔSET^).
7. Heatmaps showing the H3K36me3 enrichment density (left) and corresponding transcriptional profiles (right) of CWDE genes.
