# Supplementary material for: The Methyltransferase CcKmt3 Regulates Cell Wall Degradation Enzymes Activity to Enhance the Infection Process in Cytospora chrysosperma
Source: Mol Plant Pathol. 2026 Apr 1;27(4):e70246. doi: 10.1111/mpp.70246 (PMC13045292; doi:10.1111/mpp.70246)
Supplement: Supplementary file 5 — Figure S5: Functional characterisation of CcLac11 and its role in fungal development, growth and pathogenicity in Cytospora chrysosperma . [file MPP-27-e70246-s004.docx]

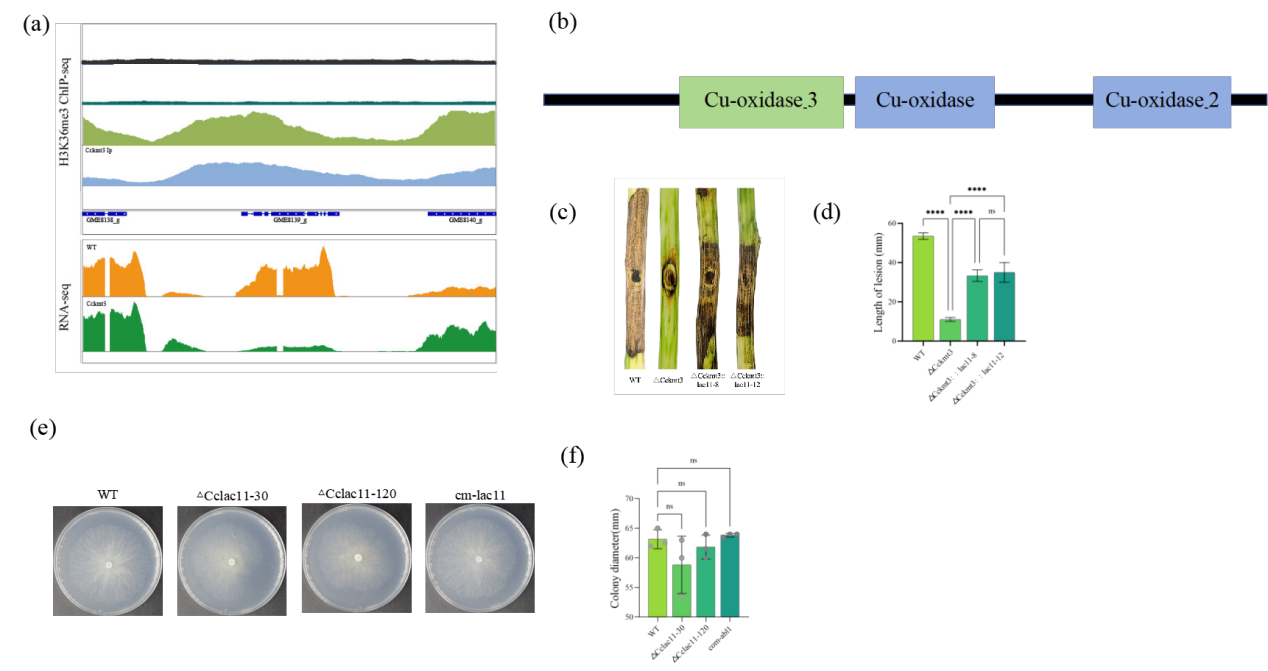


**Supplementary FIRGRE 5 Functional characterization of CcLac11 and its role in fungal development, growth, and pathogenicity in *C. chrysosperma*.**

1. Genome browser visualization of H3K36me3 signals at the *CcLac11* locus in WT and Δ*Cckmt3* strains, illustrating the impact of CcKmt3 deletion on histone modification at this gene.
2. Domain structure analysis of the CcLac11 protein, identifying functional regions associated with its activity.
3. Pathogenicity assay of the WT, Δ*Cckmt3*, Δ*Cclac11*, and Δ*Cckmt3*::Lac11 strains on poplar branch.
4. Measurement of lesion lengths on branches at 6 dpi.
5. Colony morphology of WT, Δ*Cclac11* and com-lac11 strains cultured on PDA at 48 h.
6. Quantitative comparison of hyphal growth diameter among WT, Δ*Cclac11* and com-lac11strains.
