# Supplementary material for: The Methyltransferase CcKmt3 Regulates Cell Wall Degradation Enzymes Activity to Enhance the Infection Process in Cytospora chrysosperma
Source: Mol Plant Pathol. 2026 Apr 1;27(4):e70246. doi: 10.1111/mpp.70246 (PMC13045292; doi:10.1111/mpp.70246)
Supplement: Supplementary file 6 — Figure S6: Impact of laccase inhibitor treatment on mycelial growth in comparison to the untreated control. [file MPP-27-e70246-s008.docx]

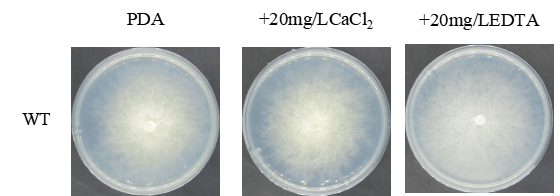


**Supplementary FIRGRE 6** Impact of laccase inhibitor treatment on mycelial growth in comparison to the untreated control.
