# Supplementary material for: The Methyltransferase CcKmt3 Regulates Cell Wall Degradation Enzymes Activity to Enhance the Infection Process in Cytospora chrysosperma
Source: Mol Plant Pathol. 2026 Apr 1;27(4):e70246. doi: 10.1111/mpp.70246 (PMC13045292; doi:10.1111/mpp.70246)
Supplement: Supplementary file 7 — Figure S7: Functional characterisation of CcPme5 and its role in fungal development, growth and pathogenicity in Cytospora chrysosperma . [file MPP-27-e70246-s009.docx]

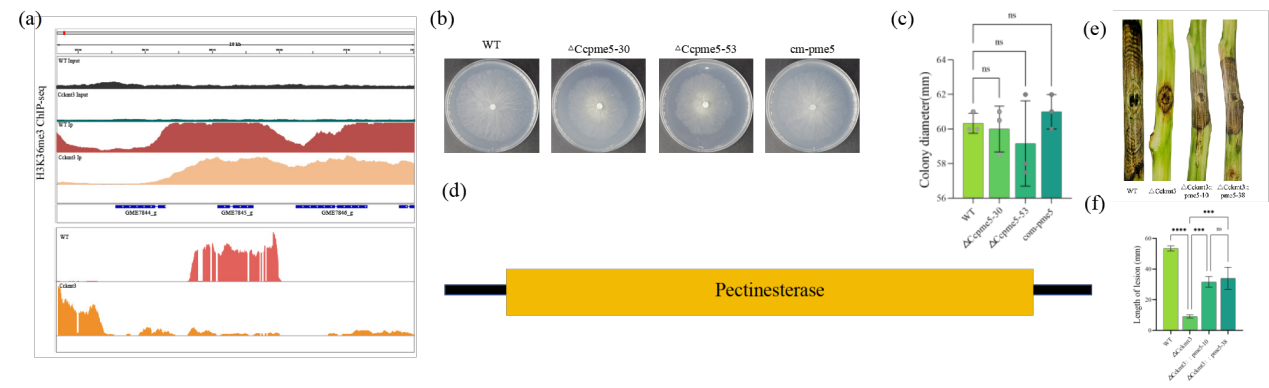


**Supplementary FIRGRE 7 Functional characterization of CcPme5 and its role in fungal development, growth, and pathogenicity in *C. chrysosperma*.**

1. Genome browser view visualization of H3K36me3 signals at the *CcPme5* locus in WT and Δ*Cckmt3* strains, illustrating changes in histone modification patterns associated with the deletion of CcKmt3.
2. Colony morphology of the WT, Δ*Ccpme5* and com-pme5 strains cultured on PDA at 48 h.
3. Comparison of hyphal growth diameter.
4. Domain structure analysis of the CcPme5 protein was conducted to identify functional regions associated with its activity.
5. Pathogenicity assay of WT, Δ*Cckmt3*, Δ*CcPme5,* and Δ*Cckmt3*::*Pme5* on poplar branches.
6. Measurement of lesion lengths on branches at 6 dpi.
