# Supplementary material for: The Methyltransferase CcKmt3 Regulates Cell Wall Degradation Enzymes Activity to Enhance the Infection Process in Cytospora chrysosperma
Source: Mol Plant Pathol. 2026 Apr 1;27(4):e70246. doi: 10.1111/mpp.70246 (PMC13045292; doi:10.1111/mpp.70246)
Supplement: Supplementary file 8 — Figure S8: Heatmaps showing the H3K36me3 enrichment density (left) and corresponding transcriptional profiles (right) of effector genes. [file MPP-27-e70246-s002.docx]

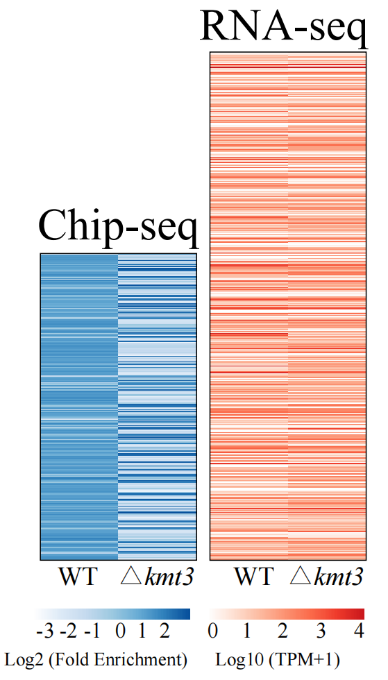


**Supplementary FIRGRE 8** Heatmaps showing the H3K36me3 enrichment density (left) and corresponding transcriptional profiles (right) of effector genes.
