# Supplementary material for: The Methyltransferase CcKmt3 Regulates Cell Wall Degradation Enzymes Activity to Enhance the Infection Process in Cytospora chrysosperma
Source: Mol Plant Pathol. 2026 Apr 1;27(4):e70246. doi: 10.1111/mpp.70246 (PMC13045292; doi:10.1111/mpp.70246)
Supplement: Supplementary file 9 — Table S1: Primers used in the study. [file MPP-27-e70246-s001.docx]

Table S1 Primers used in the study.

| **Primer name** | **Sequence** | **Purpose** |
| --- | --- | --- |
| CcKmt3-5Ffor | CATGGGCTGCTTTGAGGATC | CcSet2 5’ flanking sequence |
| CcKmt3-5Frev | CAAAGGTCGAGCAGAGAAGC |  |
| CcKmt3-3Ffor | ACATACCTTCCAAATCGCGC | CcSet2 3’ flanking sequence |
| CcKmt3-3Frev | TTCTTGTGCTGGATGAGGGT |  |
| YG-F | CGTTGCAAGACCTGCCTGAA | Hygromycin-resistance cassette |
| HY-R | GGATGCCTCCGCTCGAAGTA |  |
| External-CcKmt3for | CCCAATCTGCTCATCCGTTG | Validation of mutant deletion |
| External-CcKmt3rev | GGAGGTTCTCGGCAAAGTTC |  |
| Internal-CcKmt3for | CAAATGGCGTTCTCTACCGG | Internal sequence used for validation of mutant |
| Internal-CcKmt3rev | CCCAGAAGATCCTCCTCGTC |  |
| CcKmt3-CF | TGCTTTGCTTTCTTTCCCGG | Amplification of CcsET2 gene containing its native |
| CcKmt3-CR | AAAGAGAGGGAGAGAGGGGT |  |
| Cclac11-5Ffor | TGGACTGACTGATTGCGAGT | Cclac11 5’ flanking sequence |
| Cclac11-5Frev | GGAACGATGGCTGCTTCAAT |  |
| Cclac11-3Ffor | GGATGGGTGAGAAGGAGGAG | Cclac11 3’ flanking sequence |
| Cclac11-3Frev | GTTCGGCTCTCCTGTCTGTA |  |
| Internal-Cclac11for | TAGCTGTTCACCGGATCGAA | Internal sequence used for validation of mutant |
| Internal-Cclac11rev | CGTTGAAGGTGAGATCCCCT |  |
| External-Cclac11for | ACCACGGTAAATGTCCAGGT | Validation of mutant deletion |
| External-Cclac11rev | TATGTGAGATAACGGCGGCT |  |
| Ccpme5-5Ffor | AGGTCATTGCTGCAAAACGT | Cclac11 5’ flanking sequence |
| Ccpme5-5Frev | AGGAGCTGTAACTACCCCATG |  |
| Ccpme5-3Ffor | TGGGCAAGGTCGGGTTTATA | Cclac11 3’ flanking sequence |
| Ccpme5-3Frev | GCACATGTAGTTCCAGCCAA |  |
| Internal-Ccpme5for | CCGTCTTCAACACTACTGTCG | Internal sequence used for validation of mutant |
| Internal-Ccpme5rev | GGCGTGGTTTGAAAAGTGTG |  |
| External-Ccpme5for | ACCAAGACCCGAGTAACTGG | Validation of mutant deletion |
| External-Ccpme5rev | CGTCGATCTGGTCCTTGGAT |  |
| Ccpme5-CF | CGTGTCGTTGTTGTCCTTGT | Amplification of CcsET2 gene containing its native |
| Ccpme5-CR | ATCAGTCTCAGCCTGCACTT |  |
| RT-CcRlm1for | CGAAGATGAAGACAGCGACA | qRT-PCR of CcRlm1 |
| RT-CcRlm1rev | TCAAGGATGACAGAAACCGATC |  |
| RT-CcChs1for | GGTCTGGTTGTCTCATGGTTG | qRT-PCR of CcChs1 |
| RT-CcChs1rev | GTGGCGTACAGAAGGATCTTG |  |
| RT-CcChs5for | GCACCATCGATACCACTCTTG | qRT-PCR of CcChs5 |
| RT-CcChs5rev | GAAGGAATGGAACGAGTAGGG |  |
| RT-CcChs6for | GGAGGTGTGATCGAACTGTG | qRT-PCR of CcChs6 |
| RT-CcChs6rev | TGATGGAGGAAAGGAGGGTAG |  |
| RT-CcSet2for | ACTATTACACCAAGACCGGCA | qRT-PCR of CcSet2 |
| RT-Ccset2rev | CTTGCTGACATCCTTTGCGT |  |
| Chip-qPCR-Cclac11-1for | AAGGCTCGGTTGTCACAAAC | Chip-qPCR of Cclac11-1 |
| Chip-qPCR-Cclac11-1rev | TGGGCACTGTCTGATTGTCT |  |
| Chip-qPCR-Cclac11-2for | CGCCGTCTCATGTTCTGTTT | Chip-qPCR of Cclac11-2 |
| Chip-qPCR-Cclac11-2rev | AAGTAAGGCTGTGCTACGGA |  |
| Chip-qPCR-Ccpme5-1for | TCGTTGCAGGTTTCGAATACT | Chip-qPCR of Ccpme5-1 |
| Chip-qPCR-Ccpme5-1rev | AGGTGCTTCTGGGCTCATG |  |
| Chip-qPCR-Ccpme5-2for | TGACTTAGGTAGCTACTGGTTCC | Chip-qPCR of Ccpme5-2 |
| Chip-qPCR-Ccpme5-2rev | TGGCTTATGATCACTTTCAGGT |  |
