# Supplementary material for: The Methyltransferase CcKmt3 Regulates Cell Wall Degradation Enzymes Activity to Enhance the Infection Process in Cytospora chrysosperma
Source: Mol Plant Pathol. 2026 Apr 1;27(4):e70246. doi: 10.1111/mpp.70246 (PMC13045292; doi:10.1111/mpp.70246)
Supplement: Supplementary file 10 — Table S2: Significant differences in gene expression levels of cell wall degradation enzyme families and peak analysis. [file MPP-27-e70246-s006.docx]

**Table S2 Significant differences in gene expression levels of cell wall degradation enzyme families and peak analysis.**

| Gene ID | WT vs kmt3 significant | H3K36me3 modification mark |
| --- | --- | --- |
| GME252_g | down | peak |
| GME7084_g | down |  |
| GME8139_g | down | peak |
| GME1885_g | down |  |
| GME3937_g | down |  |
| GME5627_g | down |  |
| GME224_g | down | peak |
| GME6023_g | down |  |
| GME6086_g | down |  |
| GME7845_g | down |  |
| GME8387_g | down | peak |
| GME2022_g | down | peak |
| GME10412_g | down |  |
| GME2503_g | down |  |
| GME10186_g | down |  |
| GME9777_g | down |  |
| GME8492_g | down | peak |
| GME1890_g | down | peak |
| GME7001_g | down | peak |
| GME626_g | down |  |
| GME8433_g | down |  |
| GME7919_g | down | peak |
| GME2635_g | down | peak |
| GME2276_g | down |  |
| GME1323_g | down |  |
| GME1305_g | down |  |
| GME9377_g | down |  |
| GME2250_g | down |  |
| GME4015_g | down | peak |
| GME5886_g | down |  |
| GME1949_g | down |  |
| GME7728_g | down |  |
| GME5807_g | down |  |
| GME424_g | down |  |
| GME4165_g | down |  |
| GME3725_g | down | peak |
| GME9985_g | down |  |
| GME1518_g | down |  |
| GME7060_g | down |  |
| GME3368_g | down |  |
| GME171_g | down | peak |
| GME119_g | down |  |
| GME3580_g | down | peak |
| GME1612_g | down |  |
| GME472_g | down | peak |
| GME1315_g | down |  |
| GME7629_g | down |  |
| GME1310_g | down |  |
| GME9734_g | down |  |
| GME10288_g | down |  |
| GME5871_g | down |  |
| GME5811_g | down |  |
| GME5812_g | down |  |
| GME409_g | down | peak |
| GME411_g | down |  |
| GME412_g | down |  |
| GME413_g | down |  |
| GME414_g | down |  |
| GME415_g | down |  |
| GME417_g | down |  |
| GME418_g | down |  |
| GME419_g | down | peak |
| GME420_g | down | peak |
| GME423_g | down |  |
| GME425_g | down | peak |
| GME426_g | down | peak |
| GME428_g | down | peak |
| GME434_g | down | peak |
| GME437_g | down | peak |
| GME440_g | down | peak |
| GME7991_g | down |  |
| GME9493_g | down |  |
| GME5549_g | down |  |
| GME9766_g | down |  |
| GME10199_g | down |  |
| GME4315_g | down |  |
| GME639_g | down |  |
| GME9353_g | down |  |
| GME5089_g | down |  |
| GME4429_g | down |  |
| GME6124_g | down |  |
| GME4514_g | down |  |
| GME9609_g | down |  |
| GME110_g | down |  |
| GME6545_g | down | peak |
| GME7446_g | down |  |
| GME10366_g | down |  |
| GME8051_g | down |  |
| GME4753_g | down |  |
